# Cost-efficient behavioral modeling of antennas by means of global sensitivity analysis and dimensionality reduction

**DOI:** 10.1038/s41598-025-87465-y

**Published:** 2025-01-30

**Authors:** Slawomir Koziel, Anna Pietrenko-Dabrowska

**Affiliations:** 1 Engineering Optimization & Modeling Center, Reykjavik University, 101 Reykjavik, Iceland; 2 Faculty of Electronics, Telecommunications and Informatics, Gdansk University of Technology, 80-233 Gdansk, Poland

**Keywords:** Antenna design, Behavioral modeling, Global sensitivity analysis, Spectral analysis, Dimensionality reduction, EM-driven design, Engineering, Electrical and electronic engineering

## Abstract

Computational tools, particularly electromagnetic (EM) solvers, are now commonplace in antenna design. While ensuring reliability, EM simulations are time-consuming, leading to high costs associated with EM-driven procedures like parametric optimization or statistical design. Various techniques have been developed to address this issue, with surrogate modeling methods garnering particular attention due to their potential advantages. One key benefit is the promise of unprecedented acceleration in handling design problems that require repetitive system evaluations. However, behavioral modeling of antennas is an intrinsic endeavor. Challenges include the curse of dimensionality and the high nonlinearity of antenna characteristics. Moreover, design utility necessitates that the models are defined across wide ranges of frequency, geometry dimensions, and material parameters, posing a significant bottleneck for existing modeling frameworks. This paper introduces an innovative approach to constructing design-ready behavioral surrogates for antenna structures. Our methodology involves a rapid global sensitivity analysis (GSA) algorithm developed to determine a set of parameter space directions that maximize antenna response variability. The latter are obtained from spectral analysis of the GSA-based sensitivity indicators, and employed to define a reduced-dimensionality domain of the metamodel. The dependability of the model constructed in such a domain is superior over conventional surrogates while being suitable for design purposes. These benefits have been conclusively showcased using several microstrip antennas and illustrated by a number of design scenarios involving antenna geometry optimization for a variety of performance specifications.

## Introduction

Over the years, the technical complexity of antenna systems has increased in response to industry demands for various functionalities, such as MIMO operation, tunability, circular polarization, and others^1–4^, as well as performance requirements imposed on electrical and field properties (gain^5^, broadband operation^6^, impedance matching^7^, axial ratio^8^, radiation pattern^9^) but also geometry itself (compact size^10–13^). Accurate assessment of complex geometric devices can be effectively conducted through EM simulation, as opposed to simpler models, which, if available, often overlook critical phenomena like mutual coupling^14^, feed radiation^15^, substrate anisotropy, or the effects of connectors^16^, radomes^17^, or proximity of environmental components (e.g., human body^18^). For the same reasons, EM simulation tools have become essential at all antenna design phases (architecture evolution^19^, parametric analysis^20^, geometry parameter adjustment^21^). Auxiliary representations, such as equivalent network models, are mainly used to explain antenna operation (having its geometry already established) rather than to support the design process itself^22–25^. Recently, the importance of EM-driven parameter adjustment using rigorous numerical optimization has grown enormously as a replacement of interactive methods, such as parametric studies governed by the designer’s insight. This is indispensable as only formal methods can simultaneously handle multiple parameters, objectives, and constraints, all instrumental in identifying truly optimum designs. Yet, simulation-based optimization is computationally expensive, the single most important factor that impedes its widespread employment by antenna designers. Typical search algorithms require anything from several dozen or hundreds (gradient-based^26^ or pattern search^27^ local methods), to many thousands of EM analyses (nature-inspired algorithms^28–32^, multi-criterial optimization^33–35^, statistical design^36–38^).

The cost-related bottleneck of EM-driven design fostered the development of accelerated techniques. Among a plethora available methods, one can mention utilization of adjoint sensitivities^39,40^ (as well as other approaches reducing the cost of sensitivity evaluation in gradient-based algorithms, e.g., sparse Jacobian updating schemes^41–43^, mesh deformation^44^), the employment of fast dedicated solvers^45^, response feature techniques^46–48^, cognition-driven design^49^, or dimensionality reduction methods^50^, etc. Recent years witnessed a growing interest in surrogate-assisted methods^51–55^ that find applications in both local^56^ and global^57^ parameter tuning, uncertainty quantification^58,59^, as well as multi-criterial design^59–61^. The underlying concept is to replace expensive EM evaluations by low-cost replacement models, which can be behavioral (e.g., kriging^62^, Gaussian process regression^63^, support vector machines^64^, radial basis functions^65^, neural networks^66,67^, polynomial chaos expansion^68^, etc.) or physics-based (space mapping^69,70^, response correction^71–73^). Physics-based methods construct the surrogate model by enhancing the lower-fidelity representation, such as a parameterized equivalent circuit. Surrogate models are often employed in iterative frameworks involving sequential sampling strategies^74,75^, where the prediction phase is followed by model refinement using the acquired EM data^76^. Procedures of this sort are often referred to as machine learning algorithms^77–79^.

Undoubtedly, replacing expensive computational models with fast surrogates is attractive. However, constructing design-ready metamodels that accurately represent system outputs across broad ranges of designable parameters (geometry and material) and frequencies presents significant challenges. The nonlinearity of antenna frequency responses and the curse of dimensionality^80^ make broad-range surrogate modeling impractical beyond simple structures parameterized using a few variables. Indeed, these challenges have driven the development of iterative procedures outlined in the previous paragraph. However, while machine-learning-type methods help alleviate cost-related issues by focusing on promising regions of the search space, they often compromise versatility. Changing design specifications necessitates repetitive algorithm executions and additional costs^81^. In some cases, general-purpose modeling can be enhanced by methods such as high-dimensional model representation (HDMR)^82^, least-angle regression^83^, or multi-fidelity approaches like two-stage Gaussian process regression (GPR)^84^ and co-kriging^85^. Another approach is performance-driven (or constrained) modeling^86^, addressing dimensionality-related issues by appropriately defining the model domain. The domain is set up in the areas encapsulating high-quality designs^87^. Several variations of domain-confined methods have been developed^88–90^, including nested kriging^87^, along with generalizations to variable-fidelity regimes^91^ and deep learning^92^. However, a performance-driven surrogate corresponds to a chosen set of performance figures and the associated optimality conditions^86^. Changing these necessitates rebuilding the model. Furthermore, defining the model domain requires a pre-optimized set of reference designs, which compromises the computational efficiency of model construction. However, this particular issue can be mitigated by the reference-design-free approaches^93,94^, or exploitation of sensitivity information^95^.

This paper presents an innovative method for constructing computationally-efficient design-ready surrogate models of antenna structures. Our approach entails a rapid global sensitivity analysis procedure, designed to identify parameter space directions associated with the maximum antenna response variability. A small subset of these directions, chosen to capture the majority (e.g., 90%) of response variability, defines the surrogate’s domain. By reducing dimensionality in this manner, it becomes feasible to establish an accurate behavioral model by utilizing a small subset of the points needed by traditional methods. At the same time, as the parameter ranges within the region of validity are not formally restricted (compared to full-dimensionality space), and the domain accounts for most antenna response changes, the surrogate can be effectively used for design purposes. These properties have been demonstrated using four microstrip antennas. Comparisons with the models set up is traditional (box-constrained domains) indicate significant predictive power improvement due to the implemented mechanisms. In contrast, application case studies (antenna optimization) corroborate design usefulness of the surrogates obtained using the proposed approach.

The novelty and the technical contributions of this study can be summarized as follows: (i) development of a novel rapid global sensitivity analysis (RGSA) strategy, (ii) development of a dimensionality reduction scheme based on sensitivity analysis, (iii) development of surrogate modeling procedure employing the aforementioned mechanisms, (iv) demonstrating superior reliability and remarkable computational savings achievable due to RGSA and dimensionality reduction, (v) demonstrating that computational savings are not detrimental to design utility of the surrogate. To the authors’ best knowledge, no modeling framework featuring similar characteristics has been proposed in the literature thus far.

## Surrogate modelling by fast global sensitivity analysis and dimensionality restriction

In this part of the work we introduce the proposed modelling approach. It begins with the formulation of the modelling problem in Section "Modelling task formulation". Explanation of the rapid global sensitivity analysis (RGSA) procedure developed to derive an orthonormal set of directions associated with maximum antenna response variability is elucidated in Section "Rapid global sensitivity analysis". These directions are then applied to establish the surrogate model’s domain (Section "Model domain definition by means of RGSA"). The entire modelling workflow is summarized in Section "Modelling procedure".

### Modelling task formulation

The modelling process aims at establishing a surrogate (replacement) model ***R***_*s*_(***x***). The model is to be valid within the region of interest, typically, a box-constrained parameter space *X*. We want to ensure that the surrogate-predicted antenna characteristics ***R***_*s*_(***x***) are well-aligned with those rendered by the high-resolution EM model ***R***_*f*_(***x***). Table 1 compiles the relevant notation. The predictive accuracy of the replacement model is evaluated through a suitable error metric. In this study, we utilize the relative root mean square (RMS) error, defined as ||***R***_*s*_(***x***) – ***R***_*f*_(***x***)||/||***R***_*f*_(***x***)||. The model accuracy will be estimated using the average error *E*_*aver*_, computed for an independent set of testing points {***x***_*t*_^(*k*)^}_*k* = 1, …, *Nt*_, with

1 $$E_{aver} = \frac{1}{{N_{t} }}\sum\limits_{k = 1}^{{N_{t} }} {\frac{{||{\mathbf{R}}_{s} ({\mathbf{x}}_{t}^{(k)} ) - {\mathbf{R}}_{f} ({\mathbf{x}}_{t}^{(k)} )||}}{{||{\mathbf{R}}_{f} ({\mathbf{x}}_{t}^{(k)} )||}}}$$

**Table 1:**
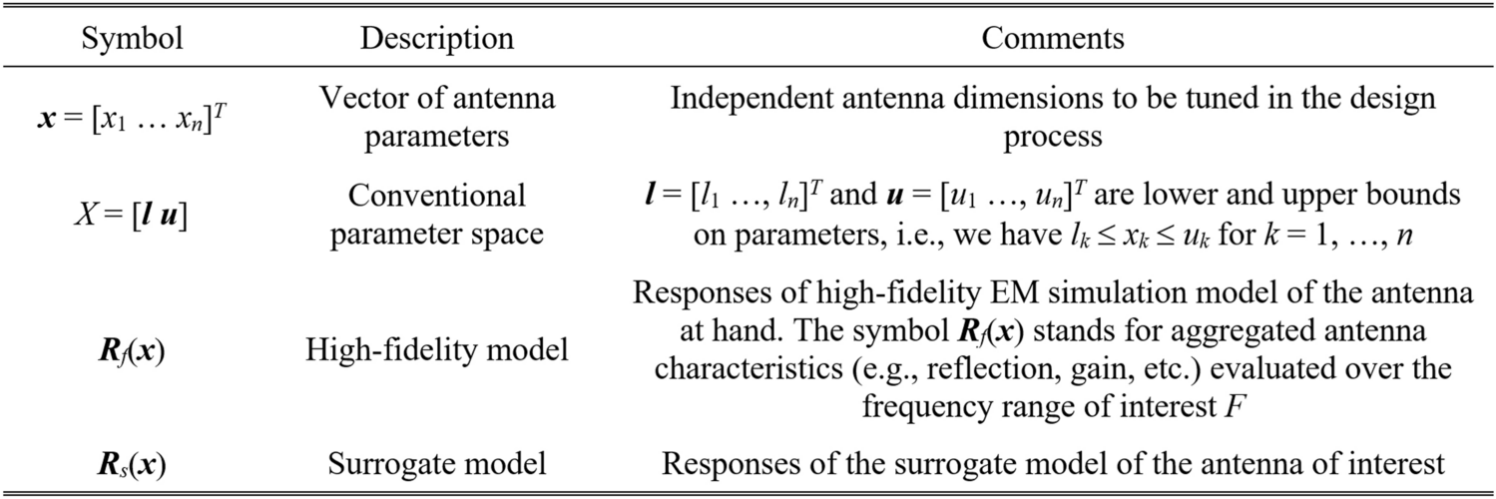
Surrogate modeling of antenna structures. Notation and terminology.

The relative error is convenient and intuitive as being independent of the actual values of the model outputs. In practical applications, a model error level of around five to eight percent is typically acceptable for design purposes. Further discussions on alternative error metrics are available in the literature (e.g.,^96,97^).

### Rapid global sensitivity analysis

As previously mentioned, the primary challenge in surrogate modeling of antenna systems stems from a combination of factors, including the curse of dimensionality, excessive ranges of geometry and material parameters the model must accommodate to ensure its utility in design, and the nonlinearity of antenna characteristics, both concerning design variables and frequency. Among these challenges, dimensionality-related issues are particularly critical. Generally speaking, assuming the behavior of antenna responses is relatively consistent across the parameter space, the predictive accuracy of a data-driven surrogate improves as the average distance between training data samples decreases. This distance is influenced by both the number of samples *N* and the space dimensionality *n*, and is proportional to (1/*N*)^1/*n*^. This relation is an extremely unfavorable one from the modeling standpoint. For example, reducing the distance twice in three-dimensional space requires an eight-fold larger training set, whereas doing the same for n = 10 requires a set that is over 1000 times larger.

The aforementioned remarks indicate that reducing the dimensionality of the problem is instrumental in improving the feasibility of surrogate modeling of antenna systems. In the context of global or quasi-global modeling, one of the possible approaches is variable screening (e.g., the Morris method^98^, Pearson correlation coefficients^99^, partial correlation coefficients^100^) or global sensitivity analysis (GSA) (e.g., Sobol indices^101^, Jansen method^102^, regression-based methods^103^), which allows for determining the relative significance of particular parameters, and to potentially exclude those that are of minor importance. Unfortunately, most of these techniques are expensive to execute, i.e., require large amounts of samples to compute sensitivity indicators. In particular, sensitivity analysis uses many random observables and design perturbations around them. The latter is acquired to obtain local sensitivity data, further incorporated into global analysis schemes. Consequently, the overall number of observables required by conventional GSA methods is large per se (e.g., many hundreds or even thousands of samples). It grows quickly with the dimensionality of the parameter space.

The second disadvantage is that GSA techniques normally allow the assessment of the significance of individual variables (e.g., their effects on the system outputs). It eventually leads to the possibility of removing the least significant ones from the problem (whether it is optimization or modeling). On the other hand, for most antenna structures, excluding individual parameters is rarely an option because the vast majority of geometry (even more material) variables play a certain role in shaping antenna responses, often through interactions with other parameters.

To address these issues, an alternative technique for global sensitivity analysis is proposed in this work, which is specifically developed to fulfill the following prerequisites:

- The CPU cost of GSA is low (e.g., not exceeding a hundred of antenna simulations);
- The analysis is carried out to determine important parameter space directions rather than identify important variables (regarding their effects on antenna response variability).

The second property allows us to determine a low-dimensional subspace of the design variable space, which is the most important from the point of view of antenna response variability, and to set up the surrogate model in this very space. Dimensionality reduction is a fundamental factor from the perspective of improving the computational efficiency of the modeling process or, equivalently, improving the model predictive power while using a considerably smaller training dataset than setting the model in the complete (full-dimensional) parameter space.

The proposed GSA approach, called rapid global sensitivity analysis (RGSA) works as follows.

1. Generate *N*_*s*_ random vectors ***x***_*s*_^(*k*)^ ∈ *X*, *k* = 1, …, *N*_*s*_, preferably in a uniform manner. Here, we use modified Latin Hypercube Sampling (LHS)^104^;
2. Acquire EM simulation data ***R***_*f*_(***x***_*s*_^(*k*)^), *k* = 1, …, *N*_*s*_;
3. For each *k* = 1, …, *N*_*s*_, find ***x***_*c*_^(*k*)^ = ***x***_*s*_^(*j*min)^ such that
  2 $$j_{\min } = \arg \mathop {\min }\limits_{\substack{ 1 \le j \le N_{s} \\ j \ne k } } \left\| {{\mathbf{x}}_{s}^{(k)} - {\mathbf{x}}_{s}^{(j)} } \right\|$$
  In other words, ***x***_*c*_^(*k*)^ is the vector closest to ***x***_*s*_^(*k*)^ in the norm sense;
4. Compute (normalized) relocation vectors.
  3 $${\varvec{v}}_{s}^{(k)} = \frac{{{\mathbf{x}}_{c}^{(k)} - {\mathbf{x}}_{s}^{(k)} }}{{\left\| {{\mathbf{x}}_{c}^{(k)} - {\mathbf{x}}_{s}^{(k)} } \right\|}}$$
  and the corresponding (normalized) response variabilities
  4 $$r_{s}^{(k)} = \frac{{{\mathbf{R}}_{f} ({\mathbf{x}}_{c}^{(k)} ) - {\mathbf{R}}_{f} ({\mathbf{x}}_{s}^{(k)} )}}{{\left\| {{\mathbf{x}}_{c}^{(k)} - {\mathbf{x}}_{s}^{(k)} } \right\|}}$$
  for *k* = 1, …, *N*_*s*_;
5. Define a *N*_*s*_ × *n* relocation matrix ***S*** as
  5 $${\mathbf{S}} = \left[ {\begin{array}{*{20}c} {r_{s}^{(1)} ({\mathbf{v}}_{s}^{(1)} )^{T} } \\ \vdots \\ {r_{s}^{{(N_{s} )}} ({\mathbf{v}}_{s}^{{(N_{s} )}} )^{T} } \\ \end{array} } \right]$$
  The rows of ***S*** represent relocation vectors normalized with respect to their importance in terms of how they affect the antenna response in the norm sense;
6. Perform spectral analysis of ***S***^105^ in order to find its eigenvectors ***e***_*j*_ (principal components) and the corresponding eigenvalues λ_*j*_, *j* = 1, …, *n*. The eigenvalues are ordered, so that λ_1_ ≥ λ_2_ ≥ … λ_*n*_.

The principal components ***e***_*j*_ form an orthonormal basis and determine the parameter space directions that have a decreasing impact on the response variability. The underlying idea is to define a reduced-dimensionality domain of the metamodel, in particular, to span it using a few (most essential) principal vectors. The number *N*_*d*_ of domain-defining vectors is determined as the smallest integer *N*_*d*_ ∈ {1, 2, …, *n*}, such that

6 $$\frac{{\sqrt {\sum\nolimits_{j = 1}^{{N_{d} }} {\lambda_{j}^{2} } } }}{{\sqrt {\sum\nolimits_{j = 1}^{n} {\lambda_{j}^{2} } } }} \ge C_{\min }$$

i.e., it is the smallest number of principal components for which the corresponding (joint) relative least-square variability is not smaller than the user-defined threshold *C*_min_. Here, we set *C*_min_ = 0.9, meaning that the selected directions should account for at least ninety percent of the overall variability.

A comment should be made concerning the choice of the threshold factor *C*_min_. As mentioned earlier, the value 0.9 means that the number of principal components selected to span the surrogate model domain collectively accounts for ninety percent of antenna response variability. While the number is arbitrary, the 90% threshold is sufficiently significant: the response variability in the space orthogonal to that spanned by the selected vectors is only 10%. At the same time, it offers a sizable reduction of the modeling problem dimensionality, as illustrated in Section "Verification case studies".

**Fig. 1:**
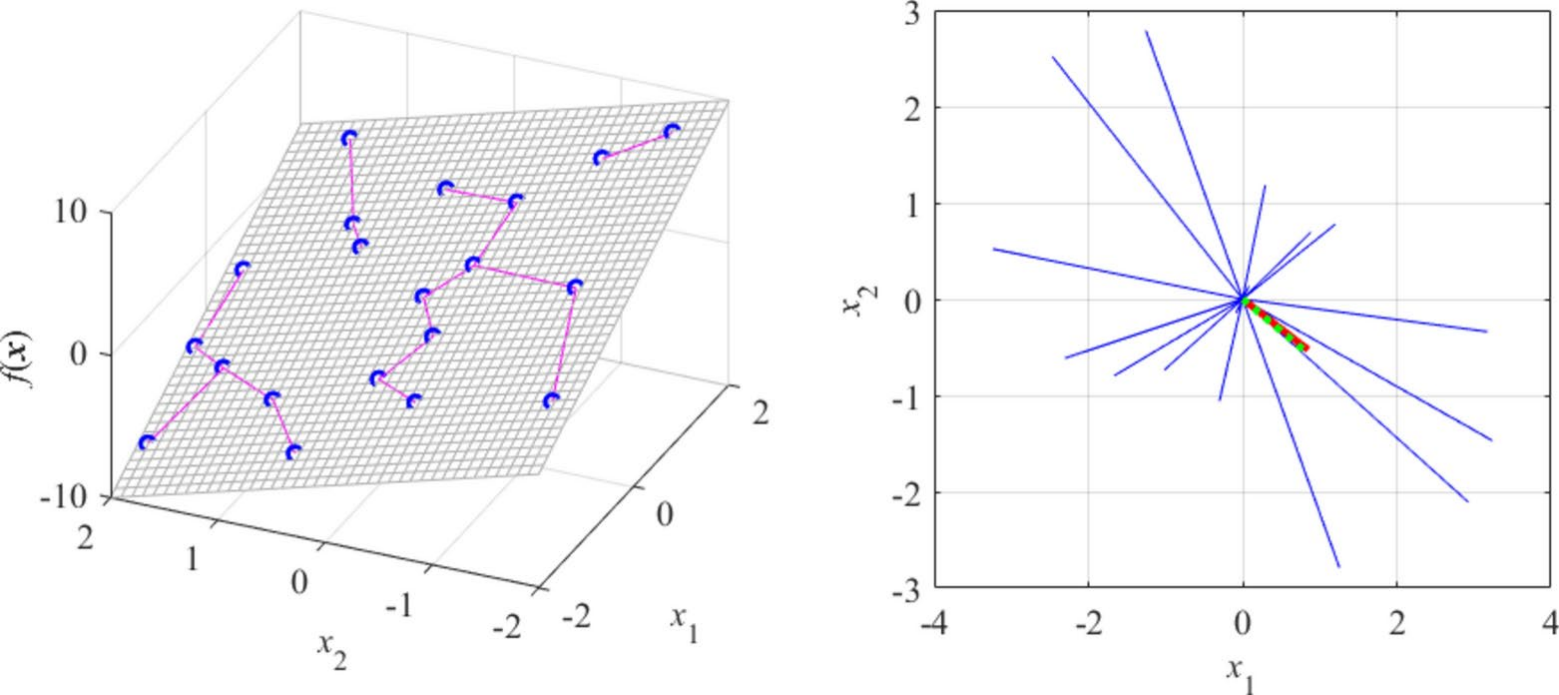
RGSA illustration using a linear function *f*(***x***) = *f*([*x*_1_ *x*_2_]^*T*^) = 3*x*_1_ – 2*x*_2_: (**a**) surface plot of the function (gray), twenty random observables ***x***_*s*_^(*k*)^ (circles), and relocation vectors ***x***_*c*_^(*k*)^ – ***x***_*s*_^(*k*)^ (line segments); (**b**) relocation matrix vectors *r*_*s*_^(*k*)^***v***_*s*_^(*k*)^ (thin lines), the largest principal component ***e***_1_ (thick solid line), and the normalized gradient ***g*** = [3–2]^*T*^/13^1/2^ (thick dotted line). In this example, all function variability occurs along the gradient ***g*** (the function is constant in the direction orthogonal to ***g***), which is well aligned with the vector ***e***_1_, obtained using the proposed RGSA.

**Fig. 2:**
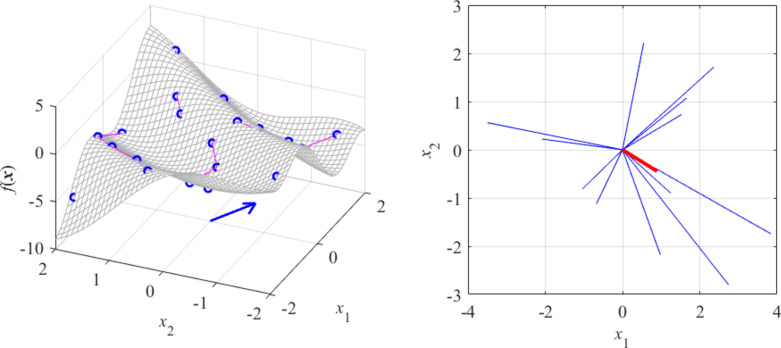
RGSA illustration using a nonlinear function of two variables: (**a**) surface plot of the function (gray), twenty random observables ***x***_*s*_^(*k*)^ (circles), and relocation vectors ***x***_*c*_^(*k*)^ – ***x***_*s*_^(*k*)^ (line segments), as well as the principal component ***e***_1_ (thick arrow); (**b**) relocation matrix vectors *r*_*s*_^(*k*)^***v***_*s*_^(*k*)^ (thin lines), and the largest principal component ***e***_1_ (thick solid line). It can be noticed that the vector ***e***_1_ obtained using RGSA visually corresponds to the direction of the largest variability of the function *f*(***x***).

Let us consider a few examples. Figure 1 shows a linear function of two variables* f*(***x***) = *f*([*x*_1_ *x*_2_]^*T*^) = 3*x*_1_ – 2*x*_2_. Here, due to linearity, the function value only changes along the gradient vector ***g*** = [3–2]^*T*^, which is confirmed by the RGSA analysis based on twenty random points. Figure 2 shows a slightly more complex situation, with the function *f*(***x***) defined so that the direction corresponding to the largest variability can be readily identified visually (as the vector perpendicular to the function ‘ripples’). Again, RGSA, based on twenty random points, correctly identified this direction.

**Fig. 3:**
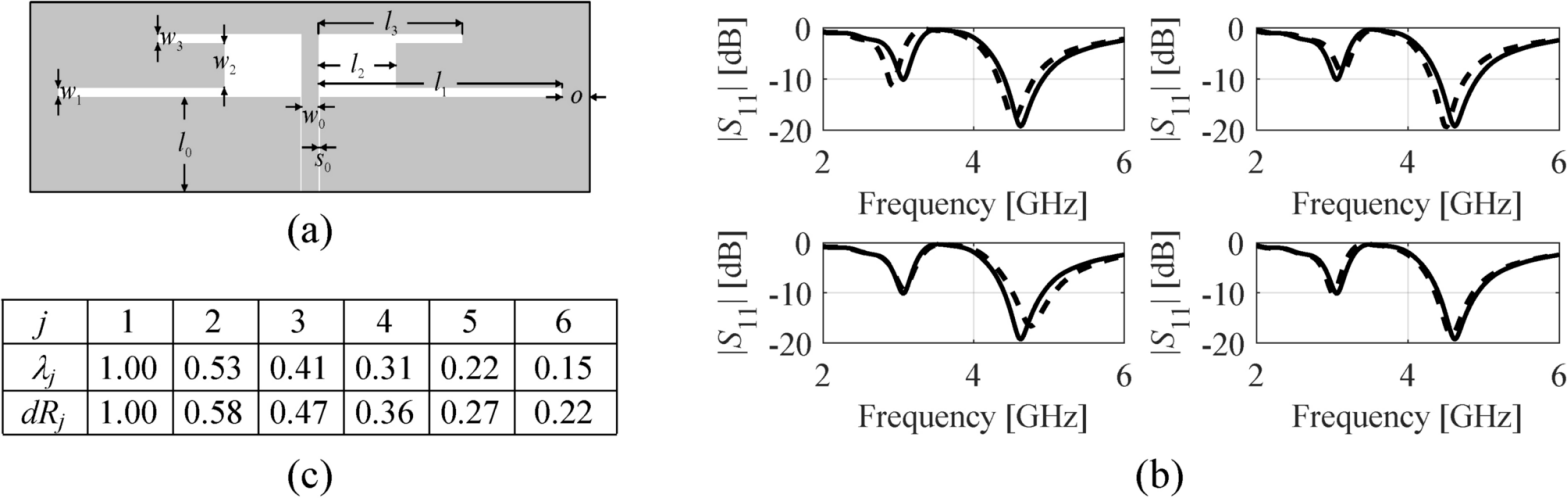
RGSA illustration using a dual-band antenna: (**a**) parameterized geometry, (**b**) |*S*_11_| responses at a random parameter vector ***x*** and designs perturbed along the principal components, ***x*** + *h****e***_*k*_ (here, *h* = 0.1) for the first four vectors (from top left to bottom right) obtained using RGSA, (**c**) normalized eigenvalues of the relocation matrix ***S*** obtained using RSGA based on fifty random samples, as well as average EM-simulated variability indicators *dR*_*j*_ computed as in (7). It can be observed that response variability is gradually reduced for increasing *k*, which demonstrates that subsequent eigenvectors correspond to directions having less and less effect on antenna responses.

Figure 3a illustrates an example of a dipole antenna parameterized with six independent variables ***x*** = [*l*_1_ *l*_2_ *l*_3_ *w*_1_ *w*_2_ *w*_3_]^*T*^. RGSA is executed based on fifty randomly allocated data points in this case.

Figure 3b illustrates antenna’s |*S*_11_| at a random parameter vector ***x*** and designs perturbed along the principal components identified using RGSA, ***x*** + *h****e***_*k*_, *k* = 1, …, *n*. As expected, the response variability is gradually reduced for *k*, increasing from 1 to *n*. For this example, we also performed statistical analysis by generating *N*_*r*_ random vectors ***x***_*r*_^(*k*)^, *k* = 1, …., *N*_*r*_ (here, *N*_*r*_ = 20), along with their perturbations ***x***_*r*_^(*k.j*)^ = ***x***_*r*_^(*k*)^ + *h****e***_*j*_, *j* = 1, …, *n*. Upon acquiring EM simulation data ***R***_*f*_(***x***_*r*_^(*k*)^), *k* = 1, …, *N*_*r*_, and ***R***_*f*_(***x***_*r*_^(*k.j*)^), *k* ∈ {1, …, *N*_*r*_}, *j* ∈ {1, …, *n*}, the variability indicators have been computed as

7 $$dR_{j} = \frac{1}{{N_{r} }}\sum\limits_{k = 1}^{{N_{r} }} {\left\| {{\mathbf{R}}_{f} ({\mathbf{x}}_{r}^{(k)} ) - {\mathbf{R}}_{f} ({\mathbf{x}}_{r}^{(k.j)} )} \right\|}$$

for *j* = 1, …, *n*, which correspond to the average response variability in directions ***e***_*j*_. Figure 3c compares the normalized eigenvalues *λ*_*j*_ and *dR*_*j*_. It should be noted that both are well aligned, which corroborates the relevance of RGSA.

Note that the CPU cost of RGSA is low. For many of the previously mentioned GSA methods (e.g., Sobol indices^101^, regression-based methods^103^) the typical number of samples required to obtain reliable sensitivity assessment is many hundreds to a few thousands for medium- to large-dimensionality problems. RGSA is executed using a few dozen random points (more specifically, fifty, in the verification experiments discussed in Section "Verification case studies"). Clearly, the sensitivity estimation may not be as accurate as for more expensive methods, yet sufficient for our purposes. Furthermore, RGSA yields principal directions that may be oriented in an arbitrary manner with respect to the coordinate system axes. Consequently, it does not eliminate any particular parameter but accounts for possible variable interactions.

### Model domain definition by means of RGSA

**Fig. 4:**
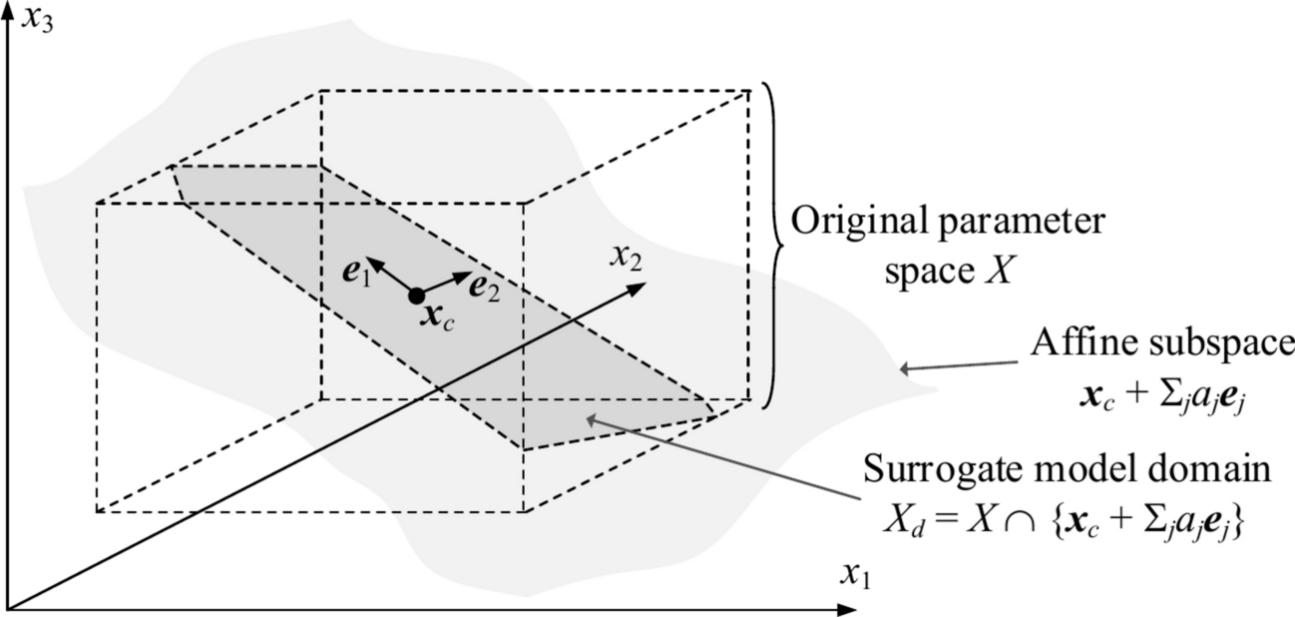
Defining reduced-dimensionality model domain *X*_*d*_. Here, the original parameter space is three dimensional, whereas *X*_*d*_ is established using two eigenvectors ***e***_1_ and ***e***_2_. Note that *X*_*d*_ is a set theory intersection of *X* and the affine subspace ***x***_*c*_ + Σ_*j*=1,2 _*a*_*j*_***e***_*j*_.

The eigenvectors generated using RGSA are used here to identify the model’s domain *X*_*d*_. The latter is spanned by the first *N*_*d*_ vectors ***e***_*j*_, *j* = 1, …, *N*_*d*_. The number of directions is determined according to (5). Formally, we have

8 $$X_{d} = \left\{ {{\mathbf{x}} \in X:{\mathbf{x}} = {\mathbf{x}}_{c} + \sum\limits_{j = 1}^{{N_{d} }} {a_{j} {\mathbf{e}}_{j} } } \right\}$$

where ***x***_*c*_ = [***l*** + ***u***]/2 is the center of the original domain *X* (cf. Table 1), and *a*_*j*_, *j* = 1, …, *N*_*d*_, are real numbers. Figure 4 shows a conceptual illustration of the set *X*_*d*_.

It is worth noting that while the dimensionality of *X*_*d*_ is lower than *n*, the domain incorporates the parameter space directions that are most critical for antenna response variability. This ensures that the surrogate model established within it is useful for design purposes.

After defining the domain, the surrogate model is established using kriging Interpolation^106^. However, the specific choice of modeling technique is of secondary importance, as our primary goal is to explore the computational benefits of dimensionality reduction achieved through RGSA.

### Modelling procedure

**Fig. 5:**
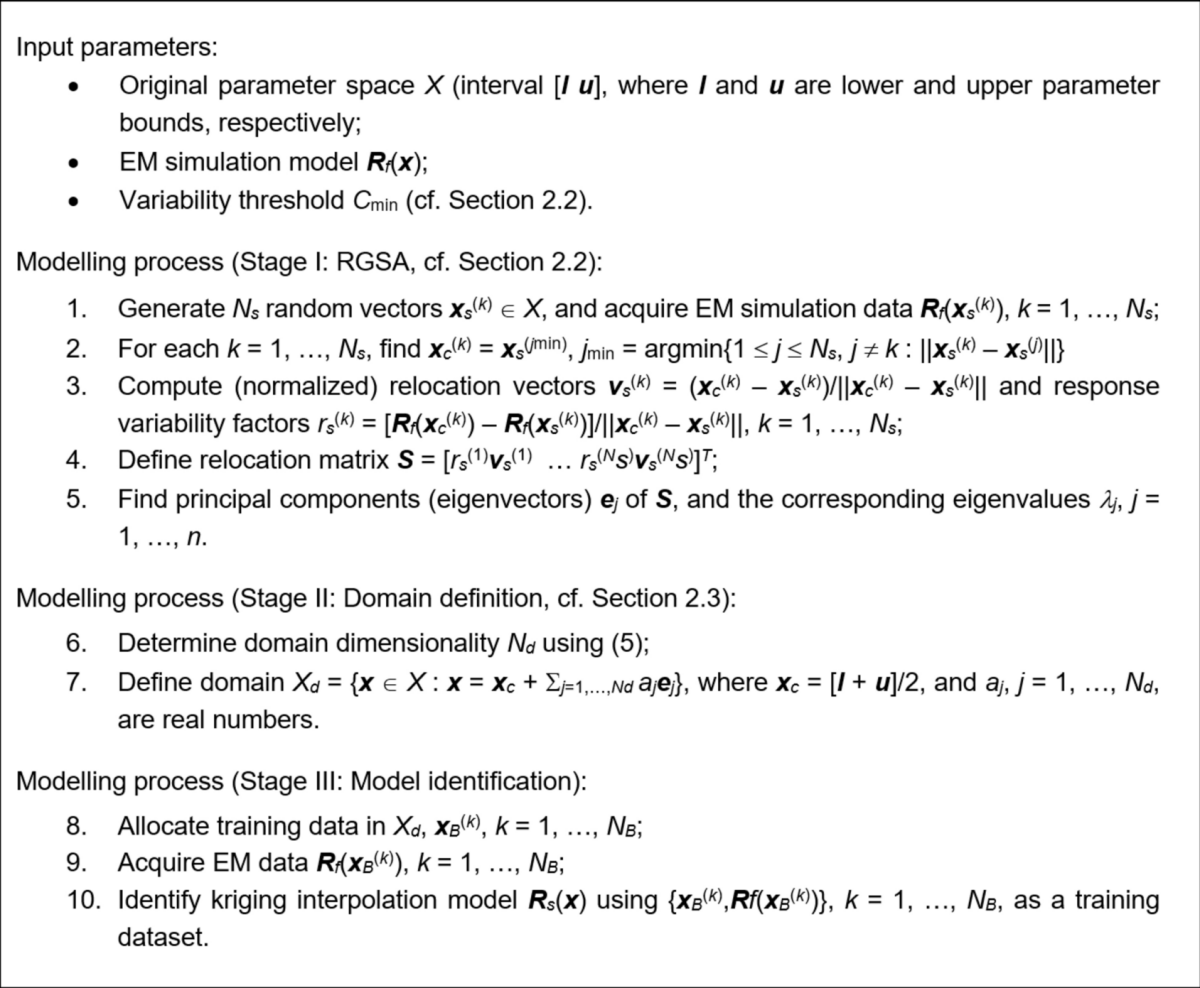
Surrogate modelling of antenna structures using RGSA and reduced-dimensionality surrogates.

**Fig. 6:**
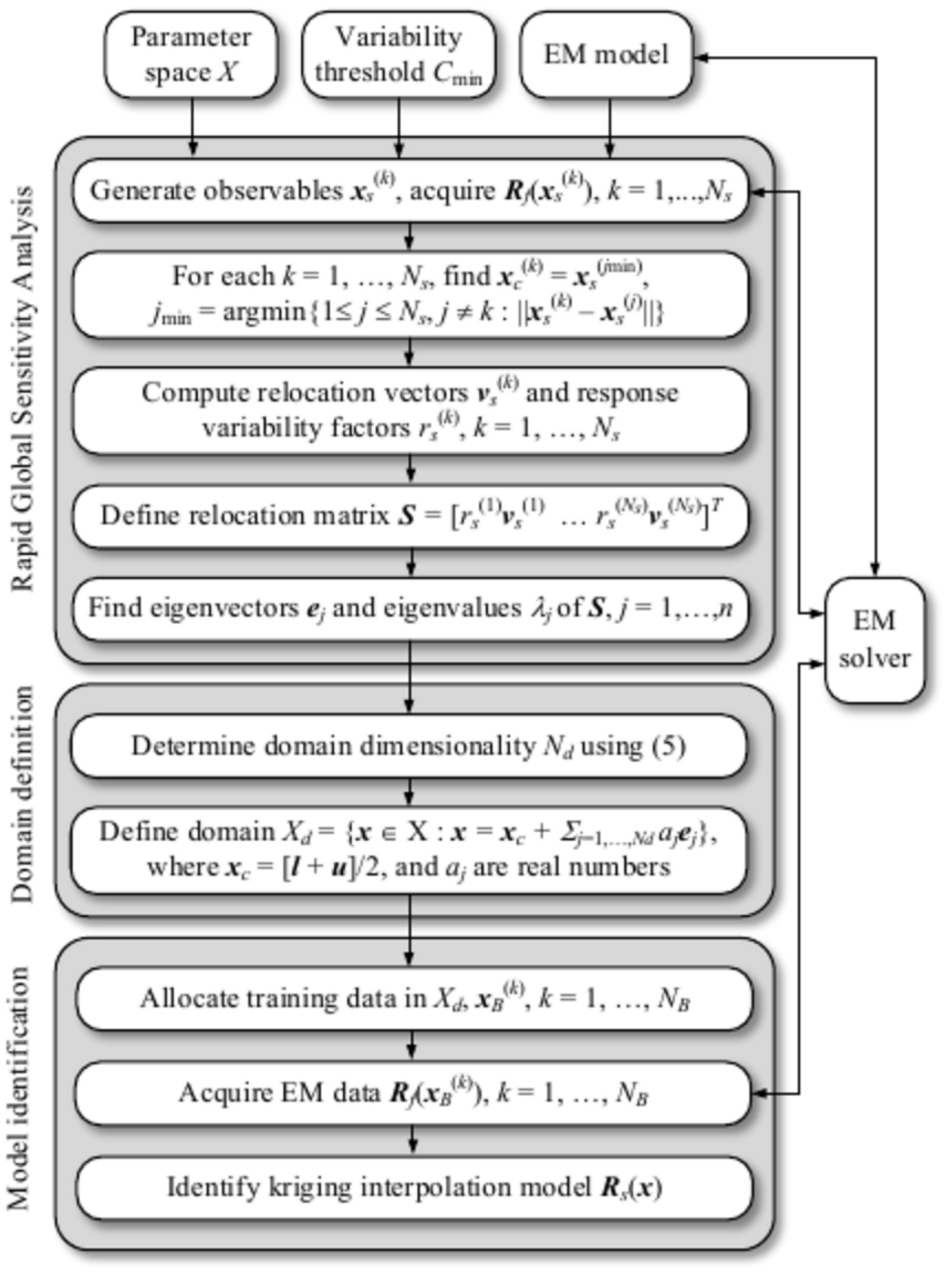
Operating flow of the suggested procedure for surrogate modeling of antennas using RGSA and reduced-dimensionality surrogates.

The entire modeling procedure is encapsulated in Fig. 5 in the form of pseudocode, delineating three distinct stages: Stage I (rapid global sensitivity analysis, RGSA), Stage II (surrogate model domain definition), and Stage III (model identification). It should be emphasized that the only control parameter of the modeling process is the variability threshold *C*_min_, which, in Section "Verification case studies", is set to 0.9. For additional clarity, Fig. 6 illustrates the flow diagram of the modelling process. The number *N*_*s*_ of random observables employed to conduct the sensitivity analysis is typically set to 50 to ensure the computational efficiency of the RGSA process. Only for test cases with the parameter space dimensionality exceeding ten or so is it increased to 100. In general, *N*_*s*_ should increase with the number *n* of design variables, and setting *N*_*s*_ = 6*n* seems to be a reasonable rule of thumb.

The number of training samples *N*_*B*_ is normally selected based on the available computational budget (note that EM simulations are generally expensive, and it is not practical to acquire large training sets). Typically, a few hundred samples are what can be afforded. In this work, we construct the surrogate models using *N*_*B*_ of various values, from 50 to 800, to investigate the scalability of the model’s predictive power as a function of the training dataset cardinality.

It should be noted that the last stage of the modeling process (Stage III) consists of three steps. The first one is allocating the training points, which is realized using Latin Hypercube Sampling to yield the normalized set of samples. These are further mapped into the dimensionality-reduced domain using an affine transformation defined using the domain-defining quantities, specifically, the center vector ***x***_*c*_ and the eigenvectors ***e***_*j*_ (cf. (8)). Subsequently, EM simulation is performed at the training designs and the kriging interpolation model is identified (through maximum likelihood estimation^106^).

## Verification case studies

Here, we present numerical verification of the proposed modeling methodology. The analysis involves four microstrip structures comprising a ring-slot antenna, a dual-band uniplanar dipole, and two quasi-Yagi antennas. The modeled characteristics include reflection coefficients and realized gain as functions of frequency. We compare the reduced-domain approach to conventional modeling using factors such as reliability and the CPU cost of establishing the surrogate, and the scalability of the modeling error as a function of the training dataset size. Additionally, we showcase design applications of the models by conducting antenna optimization across various scenarios.

### Test cases

**Fig. 7:**
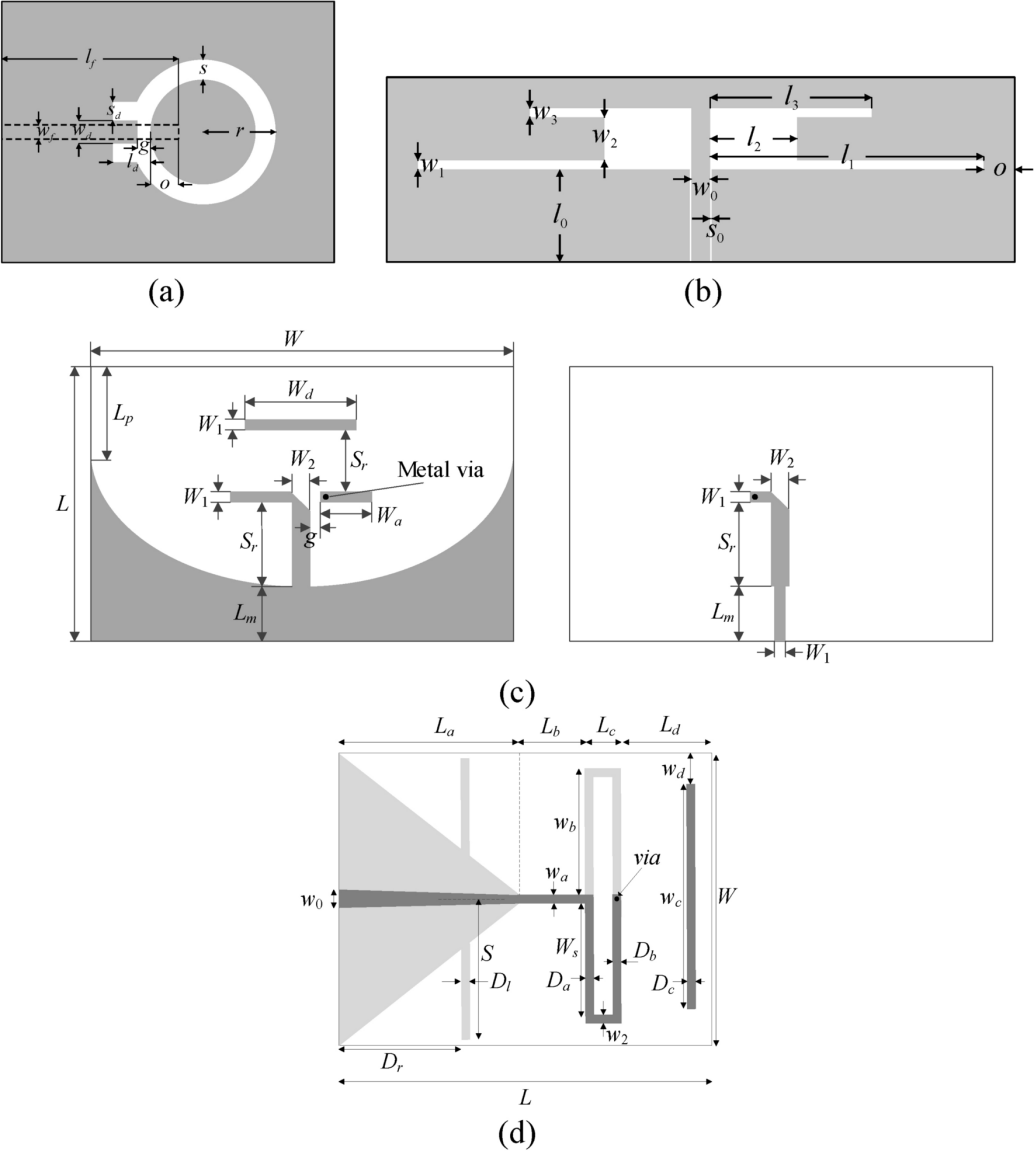
Test antennas: (**a**) Antenna I^107^, dashed line marks the microstrip feed line, (**b**) Antenna II^108^, (**c**) Antenna III^110^ (top and bottom layer shown on the left and right, respectively), (**d**) Antenna IV^110^ (top and bottom layer shown using dark- and light-grey shades).

The antennas used to demonstrate the proposed modeling procedure are shown in Fig. 7. There are four devices:

- A ring-slot antenna (Antenna I), Fig. 7a,
- A dual-band uniplanar dipole antenna (Antenna II), Fig. 7b,
- A quasi-Yagi antenna with a parabolic reflector (Antenna III), Fig. 7c,
- A quasi-Yagi antenna with integrated balun (Antenna IV), Fig. 7d.

Information about material parameters (substrate height and relative permittivity), design variables, and parameter spaces is included in Table 2. EM models of all antennas are evaluated in CST Microwave Studio (time-domain solver).

**Table 2:**
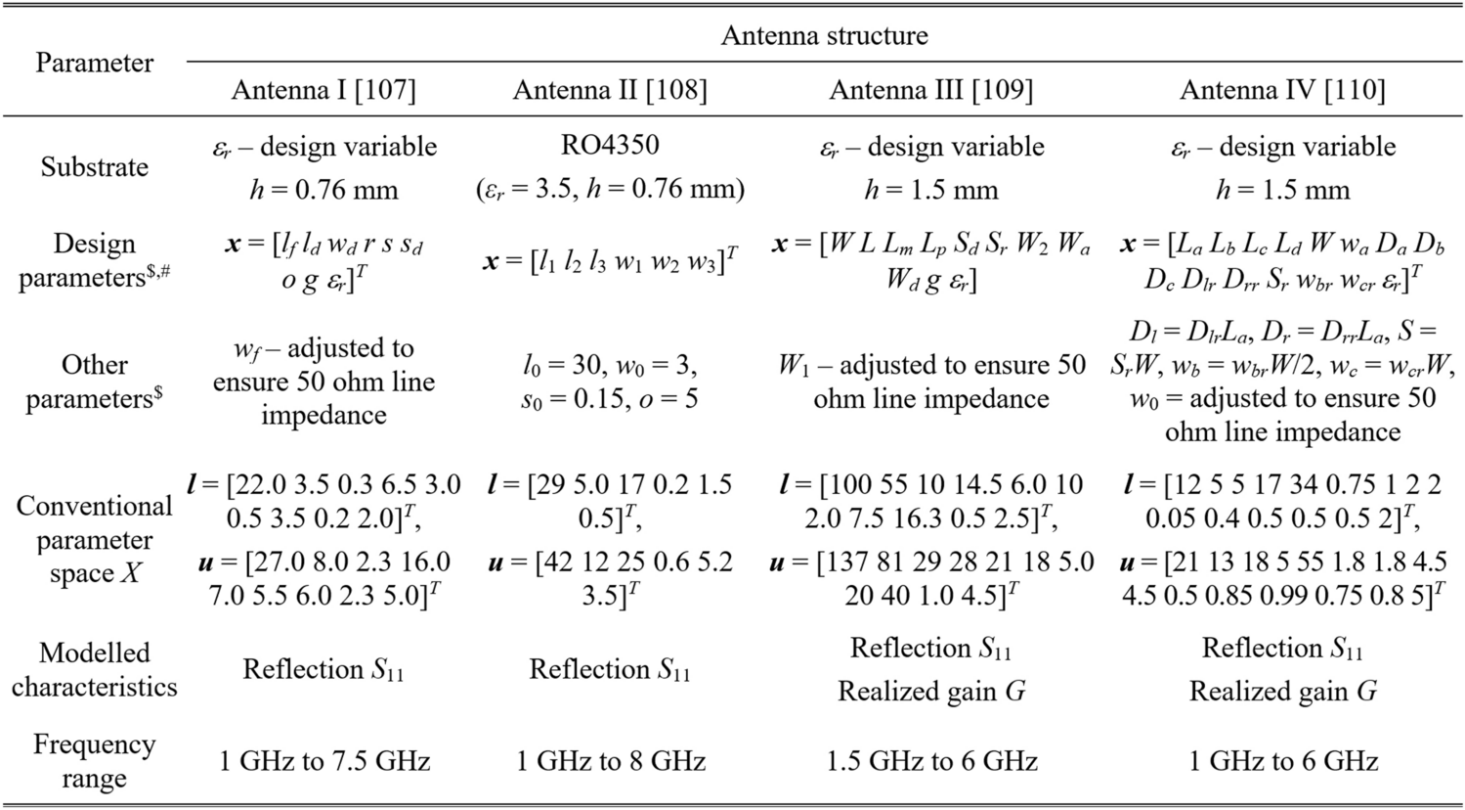
Essential parameters of verification antennas.

The considered modeling tasks are challenging. On the one hand, the parameter spaces are relatively high dimensional, from six parameters for Antenna II to fifteen for Antenna IV. Note that substrate permittivity is also included for three structures as a design parameter. On the other hand, the ranges of design variables are wide: the ratio between the upper and lower parameter bound is higher than three on average. Also, we are interested in modeling complex reflection responses and realized gain characteristics (for Antennas III and IV) all over broad ranges of frequencies as specified in Table 2, e.g., from 1 GHz to GHz for Antenna I or from 1 to 6 GHz for Antenna IV.

### Experimental setup

The verification experiments are structured to address two primary inquiries: (i) the extent to which RGSA-based modeling enhances the predictive capability of the surrogate and (ii) whether dimensionality reduction, as proposed in Section "Surrogate modelling by fast global sensitivity analysis and dimensionality restriction", impacts the design utility of the model. The essence of the second question lies in determining whether reducing the number of dimensions in the domain maintains sufficient flexibility of the metamodel for effective design purposes. To answer the first question, we compare models built in the conventional parameter space *X* with those in the confined domain. Sensitivity analysis uses fifty samples distributed in *X* via Latin Hypercube Sampling^104^. The dimensionality of the restricted domain is adjusted with *C*_min_ = 0.9 in (5), as discussed in Section "Rapid global sensitivity analysis", indicating that the domain should encompass at least ninety percent of antenna response variability. The surrogates are established using datasets of varying sizes, between 50 and 800 samples (with 1600 samples for Antenna IV, the most challenging case), allowing for an examination of model scalability. As for the second question, the RGSA-based surrogate models are utilized for antenna parameter tuning across different sets of design specifications. Given that the modeling process spans broad frequency ranges, the models can be applied to optimize antennas for various target operating bandwidths and different substrate materials (considering substrate permittivity as one of the design parameters, adjustable within a wide range from 2.0 to 5.0).

The surrogate is built using kriging interpolation with Gaussian correlation function and a trend function implemented utilizing a second-order polynomial. The model accuracy is estimated by means of a relative root mean square (RMS) error. The latter is defined as ||***R***_*s*_(***x***) – ***R***_*f*_(***x***)||/||***R***_*f*_(***x***)||, where ***R***_*s*_ and ***R***_*f*_ stand for the antenna responses predicted using the surrogate and EM analysis, respectively (cf. Section "Modelling task formulation", Eq. (1)). The error is computed using 100 randomly assigned testing vectors ***x***.

### Results

Table 3 encapsulates data on the eigenvalues *λ*_*k*_ obtained using the RGSA procedure of Section "Surrogate modelling by fast global sensitivity analysis and dimensionality restriction", and the surrogate model’s dimensionality *N*_*d*_. As mentioned earlier, *N*_*d*_ has been determined using the condition (6) with *C*_min_ = 0.9. An exception has been made for Antenna III, with *N*_*d*_ set to four and the variability factor equal to 0.89. It should be noted that the eigenvalues reduce quickly as the function of the index so that increasing the domain dimensionality by one (i.e., using *N*_*d*_ incremented by one as compared to that value obtained from (6)) does not change the antenna response variability significantly. For example, for Antenna I, the variability changes from 0.94 to 0.96 when increasing *N*_*d*_ from 4 to 5. For Antenna III, the figures are 0.89 (*N*_*d*_ = 4) and 0.92 (*N*_*d*_ = 5).

**Table 3:** RGSA data: eigenvalues *λ*_*k*_ and domain dimensionality assuming *C*_min_ = 0.9

Domain data		Antenna			
		I	II	III	IV
Dimensionality *n* of the original parameter space *X*		9	6	11	15
(Normalized) eigenvalues of the relocation matrix ***S***		*λ*_1_ = 1.00 *λ*_2_ = 0.74 *λ*_3_ = 0.63 *λ*_4_ = 0.48 *λ*_5_ = 0.35 *λ*_6_ = 0.29 *λ*_7_ = 0.23 *λ*_8_ = 0.17 *λ*_9_ = 0.06	*λ*_1_ = 1.00 *λ*_2_ = 0.53 *λ*_3_ = 0.41 *λ*_4_ = 0.31 *λ*_5_ = 0.22 *λ*_6_ = 0.14	*λ*_1_ = 1.00 *λ*_2_ = 0.88 *λ*_3_ = 0.69 *λ*_4_ = 0.60 *λ*_5_ = 0.51 *λ*_6_ = 0.41 *λ*_7_ = 0.35 *λ*_8_ = 0.32 *λ*_9_ = 0.23 *λ*_10_ = 0.20 *λ*_11_ = 0.15	*λ*_1_ = 1.00 *λ*_2_ = 0.64 *λ*_3_ = 0.57 *λ*_4_ = 0.49 *λ*_5_ = 0.37 *λ*_6_ = 0.35 *λ*_7_ = 0.29 *λ*_8_ = 0.27 *λ*_9_ = 0.22 *λ*_10_ = 0.21 *λ*_11_ = 0.18 *λ*_12_ = 0.17 *λ*_13_ = 0.13 *λ*_14_ = 0.11 *λ*_15_ = 0.06
Reduced-dimensionality domain	*N*_*d*_	4	3	4	5
	$\frac{{\sqrt {\sum\nolimits_{j = 1}^{{N_{d} }} {\lambda_{j}^{2} } } }}{{\sqrt {\sum\nolimits_{j = 1}^{n} {\lambda_{j}^{2} } } }}$	0.94	0.95	0.89	0.91

**Fig. 8:**
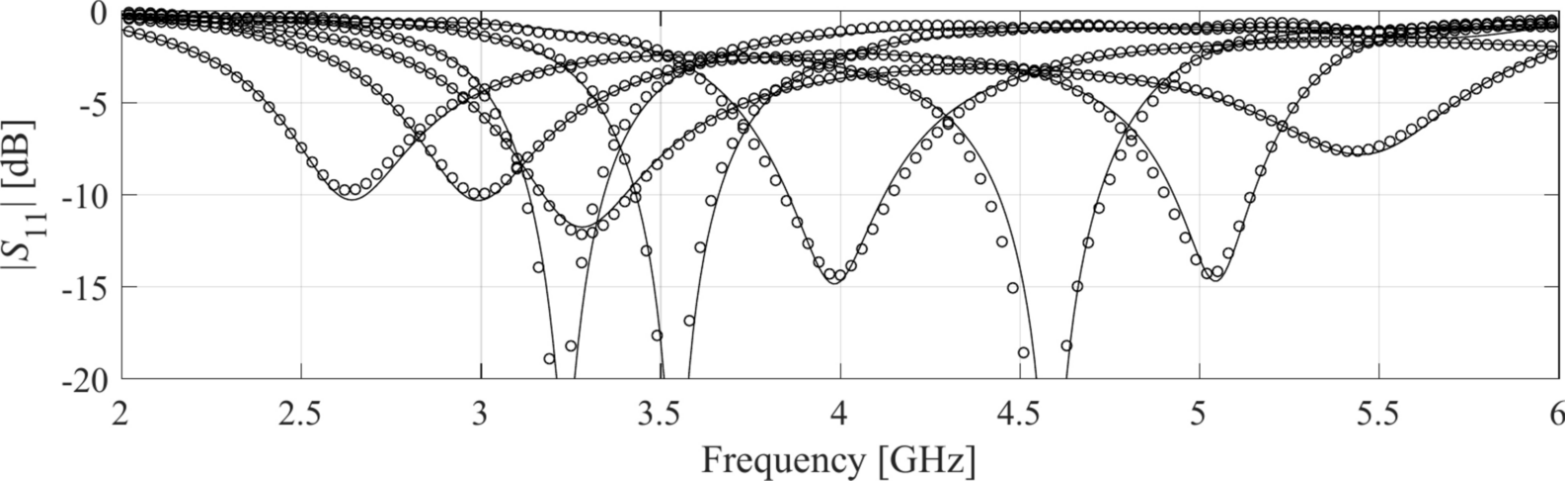
Frequency characteristics of Antenna I at the selected test points: EM simulation (—), and the prediction of the proposed surrogate (o). The metamodel constructed for *N*_*B*_ = 800.

**Fig. 9:**
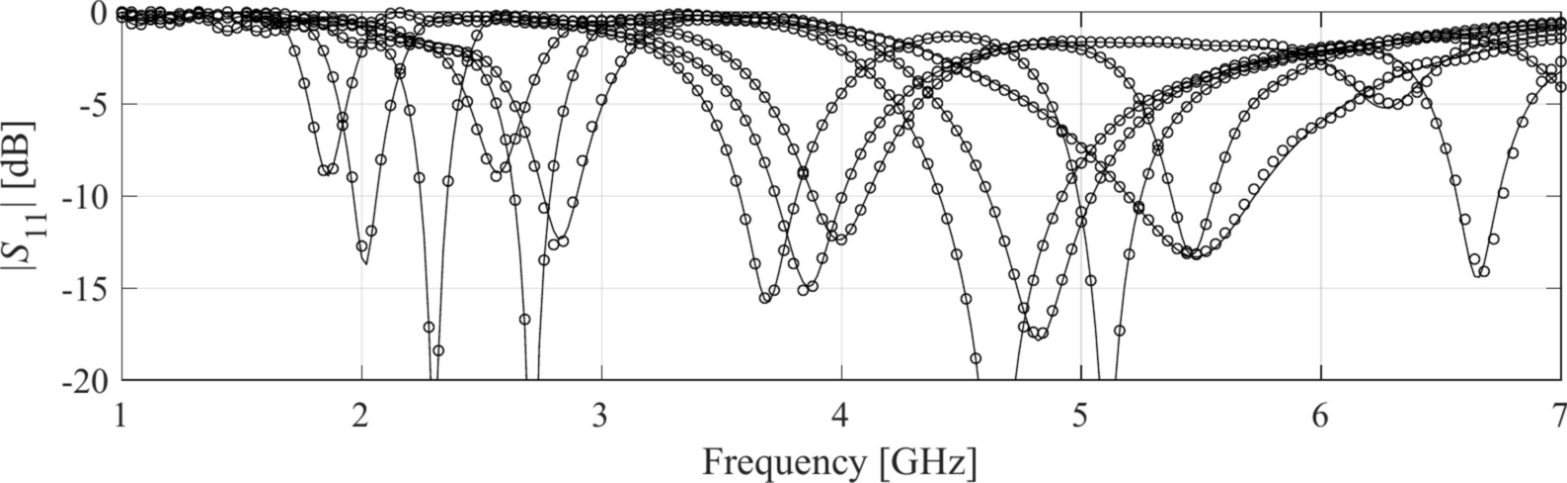
Frequency characteristics of Antenna II at the selected test points: EM simulation (—), and the prediction of the proposed surrogate (o). The metamodel constructed for *N*_*B*_ = 800.

**Fig. 10:**
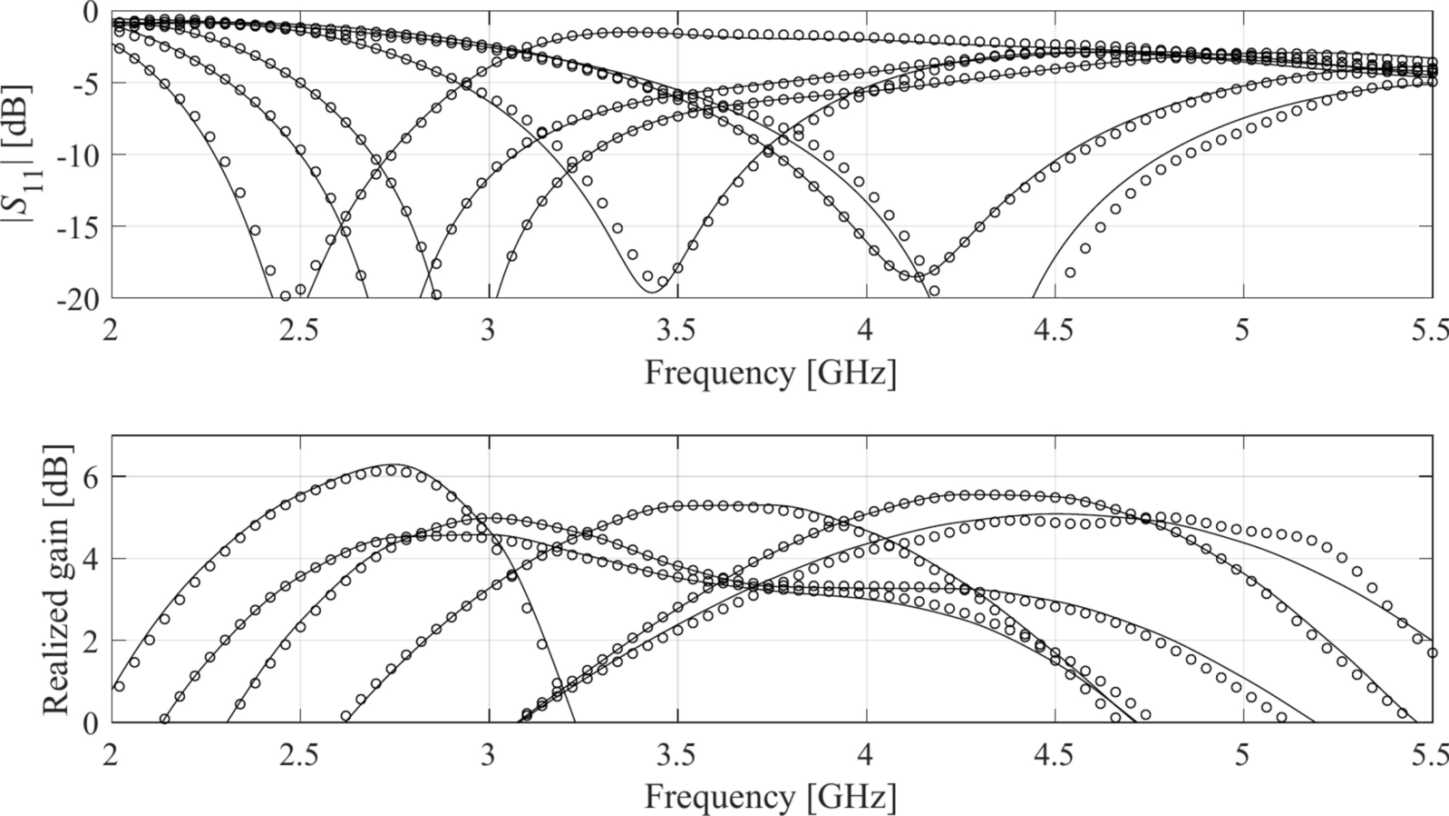
Frequency characteristics of Antenna III at the selected test points: EM simulation (—), and the prediction of the proposed surrogate (o). The metamodel constructed for *N*_*B*_ = 800.

**Fig. 11:**
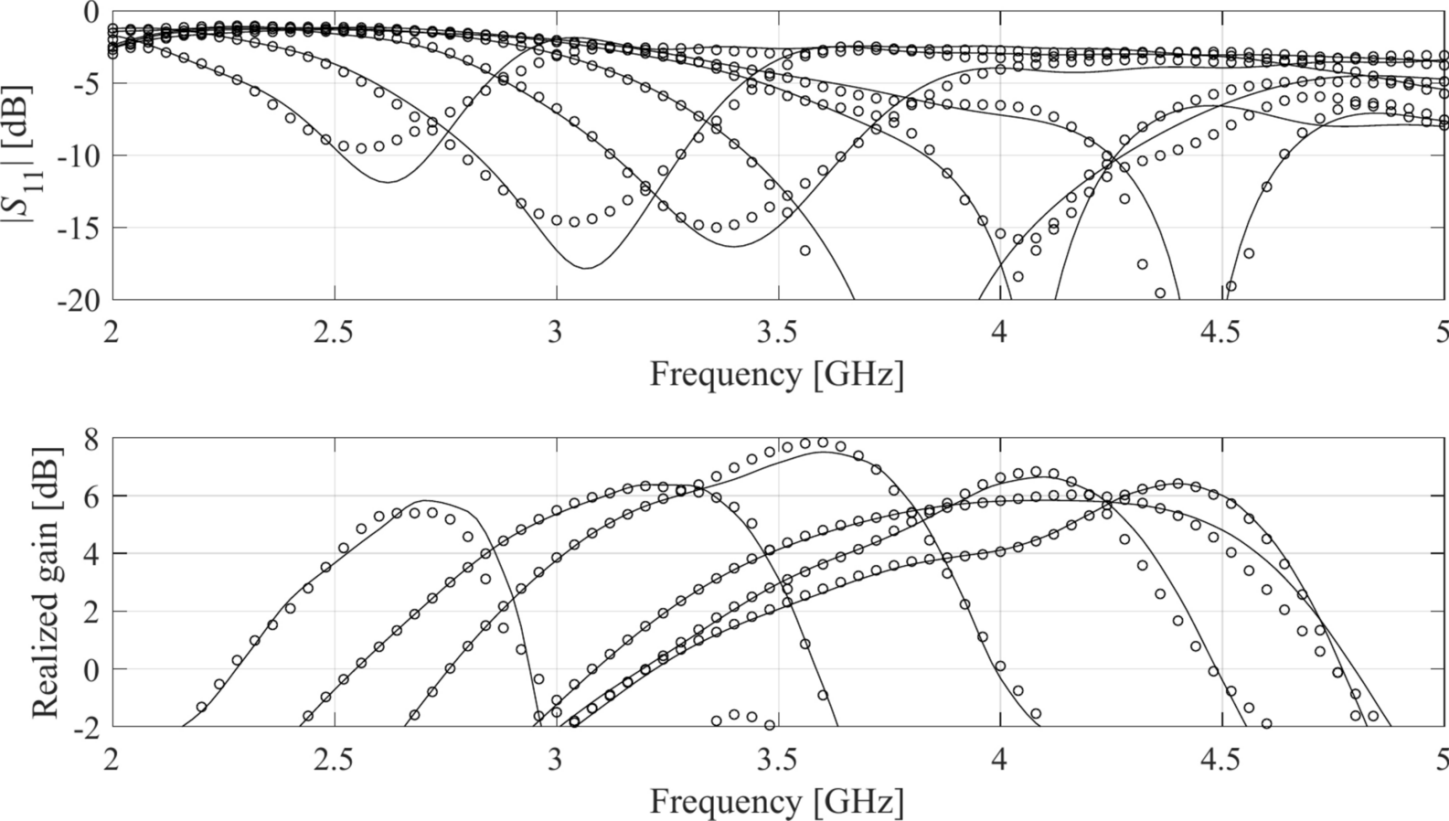
Frequency characteristics of Antenna IV at the selected test points: EM simulation (—), and the prediction of the proposed surrogate (o). The metamodel constructed for *N*_*B*_ = 1600.

Table 4 indicates the modelling errors for the surrogates built within the original space *X*, and the RGSA-based domain *X*_*d*_, for five training datasets of cardinalities 50, 100, 200, 400, and 800, respectively. As mentioned earlier, for Antenna IV, we also include a data set consisting of 1,600 samples. Antenna characteristics rendered using the metamodel and EM analysis at the chosen testing points are illustrated in Figs. 8, 9, 10, and 11 for Antennas I through IV, respectively.

**Table 4:**
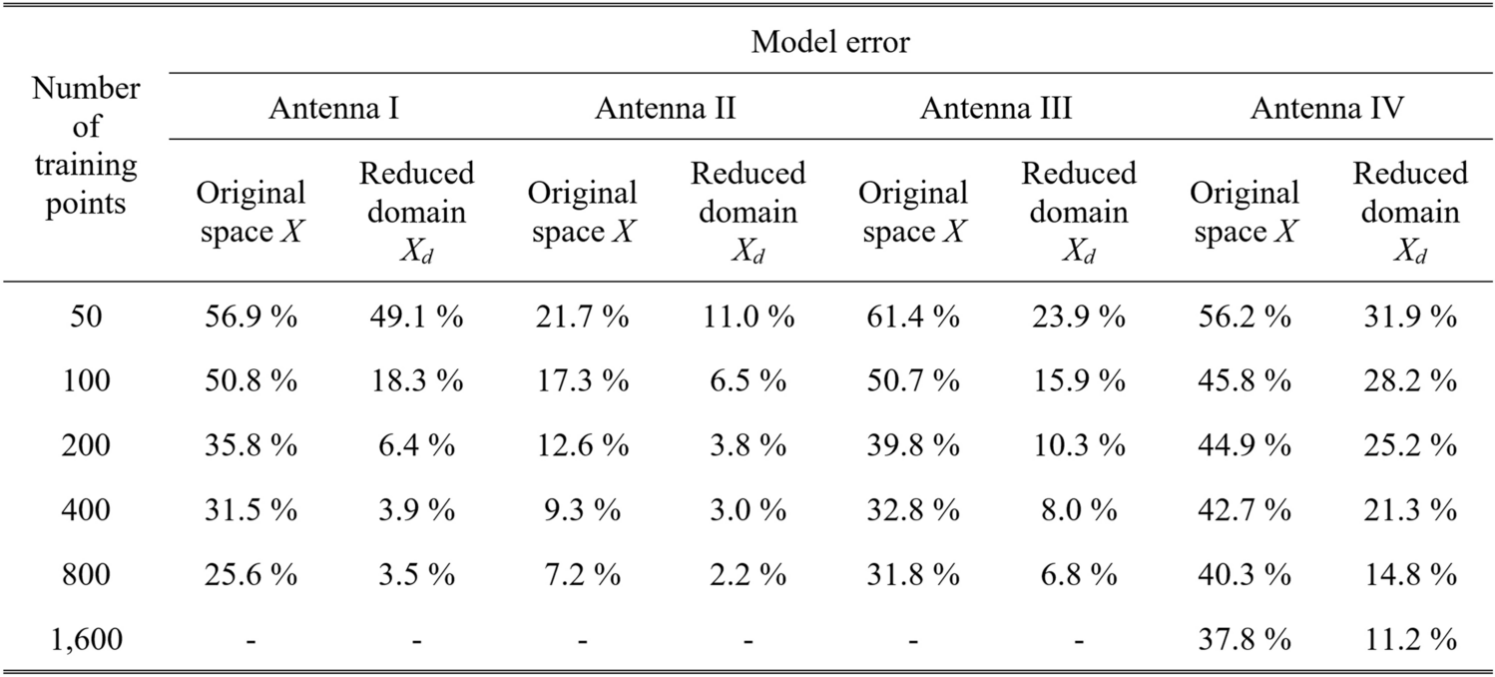
Modeling results for Antennas I through IV.

^#^When modeling in the reduced domain, the total cost of model setup also includes the samples generated for the purpose of RGSA, which are as follows: Antennas I, II, and III: 50 points; Antenna IV: 100 points.

The data in Table 4 unanimously demonstrate the computational benefits of the RGSA-based dimensionality reduction. To begin with, modeling in the original parameter space turns out to be extremely challenging. For Antenna II, the most straightforward case (six parameters), the conventional surrogate managed to secure a relative RMS error lower than ten percent, and only for the two largest datasets of 400 and 800 samples. The remaining structures’ error levels are between sixty percent (for the smallest datasets) and about thirty percent (for the largest datasets).

It should be noted that RGSA comes with some minor extra costs. As mentioned earlier, the purpose of developing RGSA was to ensure computational efficiency. The specific number of data samples utilized in our numerical experiments has been provided in the footnote of Table 4. These costs are 50 random observables for Antennas I, II, and III, and 100 observables for Antenna IV. It should be noted that Antenna IV is a complex case with 14-dimensional parameter space and broad parameter ranges therefore a larger number of RGSA samples were utilized. Nonetheless, these costs are almost negligible whenever the training dataset size exceeds 200. For example, the relative extra overhead for *N*_*B*_ = 800 is only around six percent for Antennas I, II, and III, and around twelve percent for Antenna IV. These minor expenses are traded for a dramatic improvement of the modeling reliability, as shown in Table 4.

This predictive power is not sufficient when it comes to design applications of the models. On the other hand, the proposed approach allows for significant accuracy improvement. The relative errors are as low as a few percent, which makes the surrogates suitable for solving design tasks, as discussed in Section "Application case studies". Furthermore, reduced dimensionality of the domain greatly improves the scalability of the model, i.e., enlarging the training datasets greatly affects the model’s dependability, as opposed to the conventional approach.

At this point, it should be reiterated that the major factor enabling remarkable reliability of the proposed modeling procedure is the dimensionality reduction. As mentioned in Section II.*B*, parameter space dimensionality plays a fundamental role in constructing reliable data-driven models. Assuming comparable parameter ranges, the average distance between training points (which determines modeling accuracy) scales extremely poorly with the number of antenna parameters. This means that reducing the model domain dimensionality is of fundamental importance to improve the mentioned scalability and to observe the error reduction when increasing the number of training points. Now, constructing the surrogate model domain as a reduced-dimensionality subspace embedded in the original parameter space (cf. Fig. 4 and Eq. (8)) achieves exactly that: a dramatic reliability improvement without compromising the design utility. The latter is because the domain-defining parameter space directions correspond to the largest antenna response variability. Thus, all components of the proposed modeling approach work in synergy to enable both computational efficiency and design suitability.

In terms of scalability, it can be observed that the reduction of the modeling error for conventional models when increasing the dataset size from 50 to 800 is (in terms of multiplicative factor) between 1.4 for Antenna IV to 3.0 for Antenna II. At the same time, the error reduction is from 2.2 for Antenna IV to over 14 for Antenna I. The average improvement is around two for conventional models and over six for the proposed framework. On top of this, the overall error levels are much lower for our technique, even for the smallest dataset, as already discussed earlier.

### Application case studies

Section "Results" unequivocally illustrated that reducing the domain’s dimensionality has a distinct and positive impact on the model’s predictive power and error scalability concerning the training dataset size. Here, we confirm that this reduction does not impair the model’s design utility. It is important to recall that dimensionality reduction is not aimed at eliminating specific antenna parameters; instead, it identifies directions most relevant to response variability. Therefore, the domain is expected to encompass regions that provide sufficient flexibility for shaping antenna characteristics following various design specifications imposed on the structure.

To validate this claim, our test antennas have been optimized in the sense explained in Table 5. For Antennas I and II, the primary goal is the improvement of impedance matching, whereas, for Antennas III and IV, it is the maximization of the average in-band realized gain; furthermore, a constraint is imposed on the in-band matching. Each antenna has been designed to meet four sets of specifications as specified in Tables 6, 7, 8, and 9. The same tables also contain the results regarding the geometry parameter values of the optimized antennas. Note that for Antennas I, III, and IV, substrate permittivity has been one of the design variables considered in the modeling process. This means that the same surrogate model can optimize the antenna for various substrates featuring permittivity within the prescribed range.

**Table 5:**
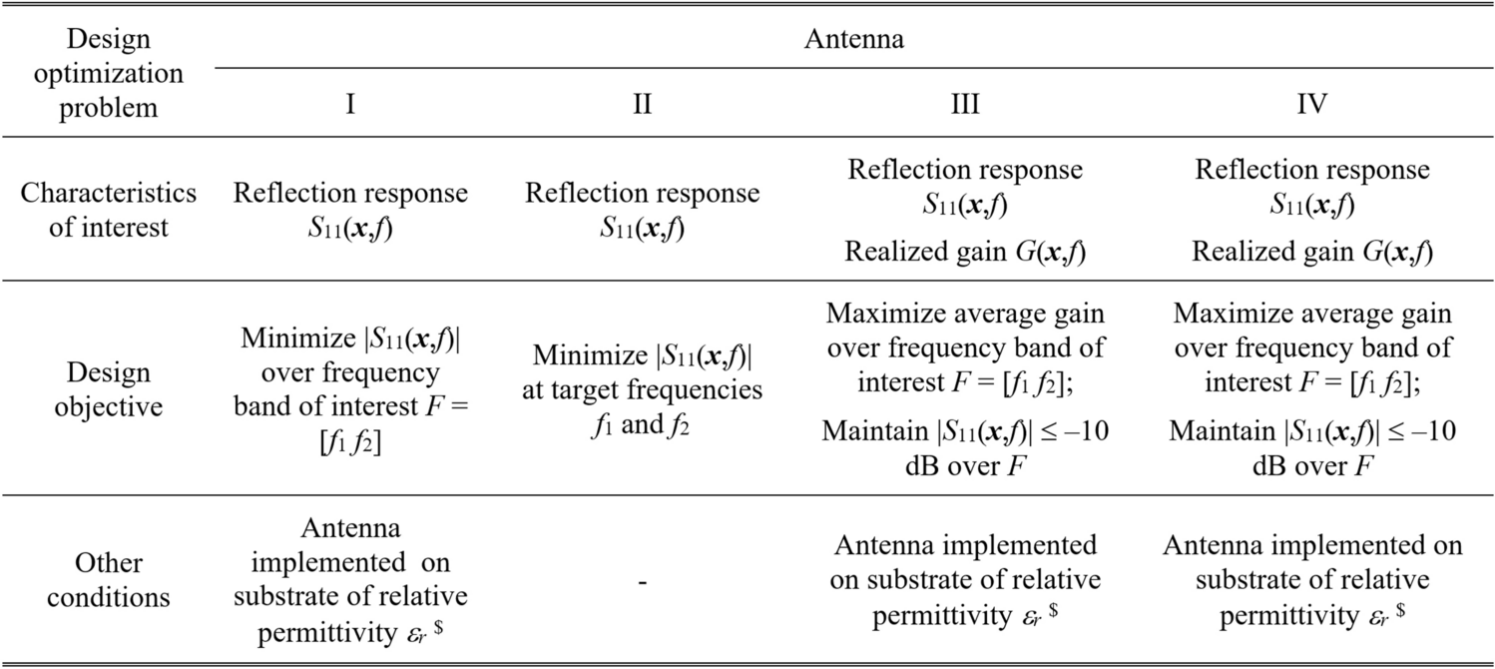
Application case studies for the considered test antennas.

**Table 6:**
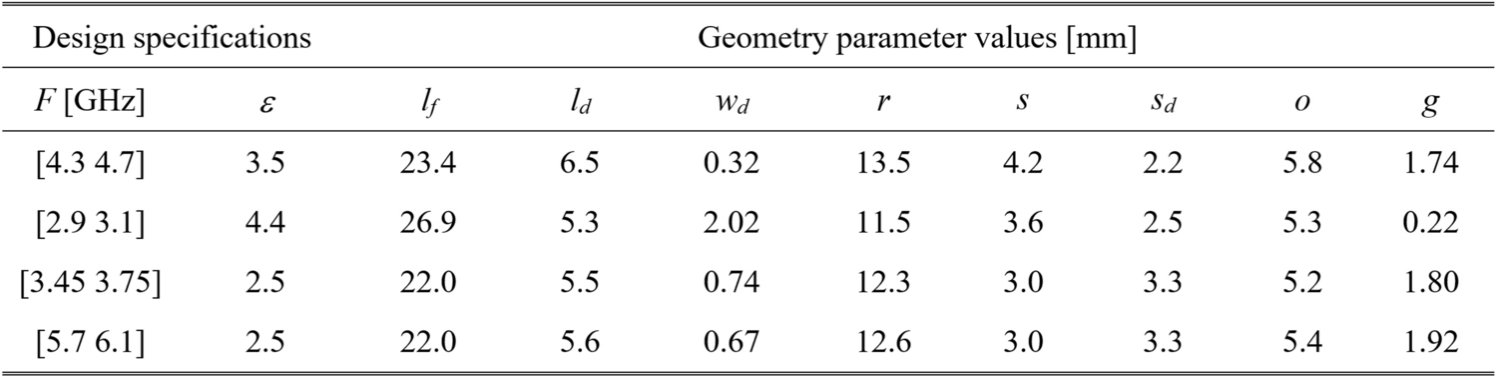
Antenna I: optimization results.

**Table 7:**
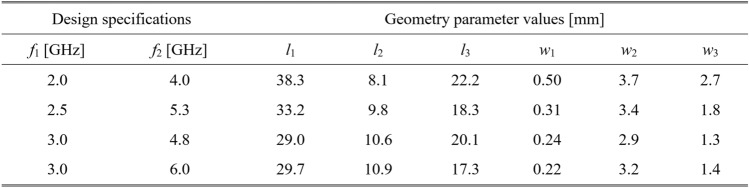
Antenna II: optimization results.

**Table 8:**
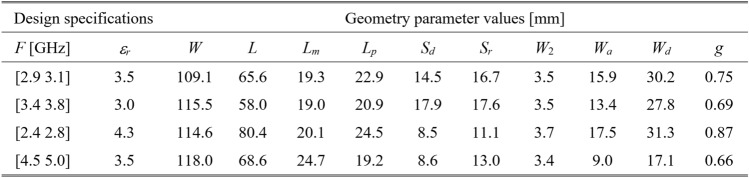
Antenna III: optimization results.

**Table 9:**
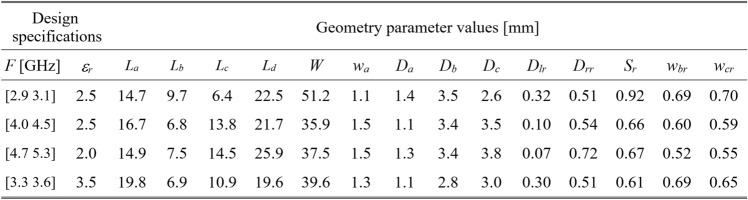
Antenna IV: optimization results.

**Fig. 12:**
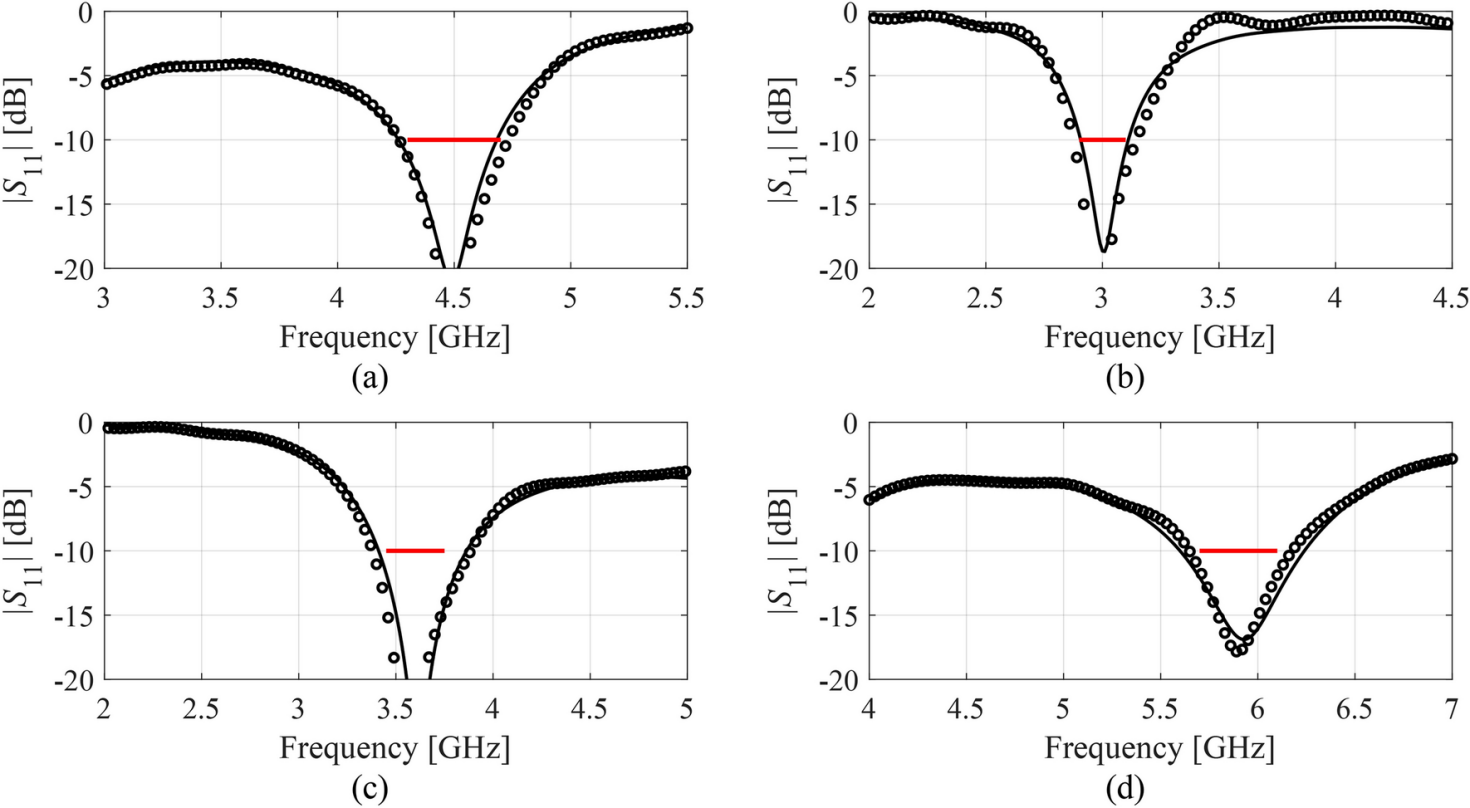
Antenna I: model-predicted |*S*_11_| at the design found by optimizing the proposed metamodel (o), and EM-evaluated characteristic (—) at the same design; model constructed for *N*_*B*_ = 800. The designs generated assuming the following specifications (bandwidth and substrate permittivity): (**a**) *F* = [4.3 4.7] GHz, *ε*_*r*_ = 3.5, (**b**) *F* = [2.9 3.1] GHz, *ε*_*r*_ = 4.4, (**c**) *F* = [3.45 3.75] GHz, *ε*_*r*_ = 2.5, (**d**) *F* = [5.7 6.1] GHz, *ε*_*r*_ = 2.5. Horizontal line marks the target operating bandwidth.

**Fig. 13:**
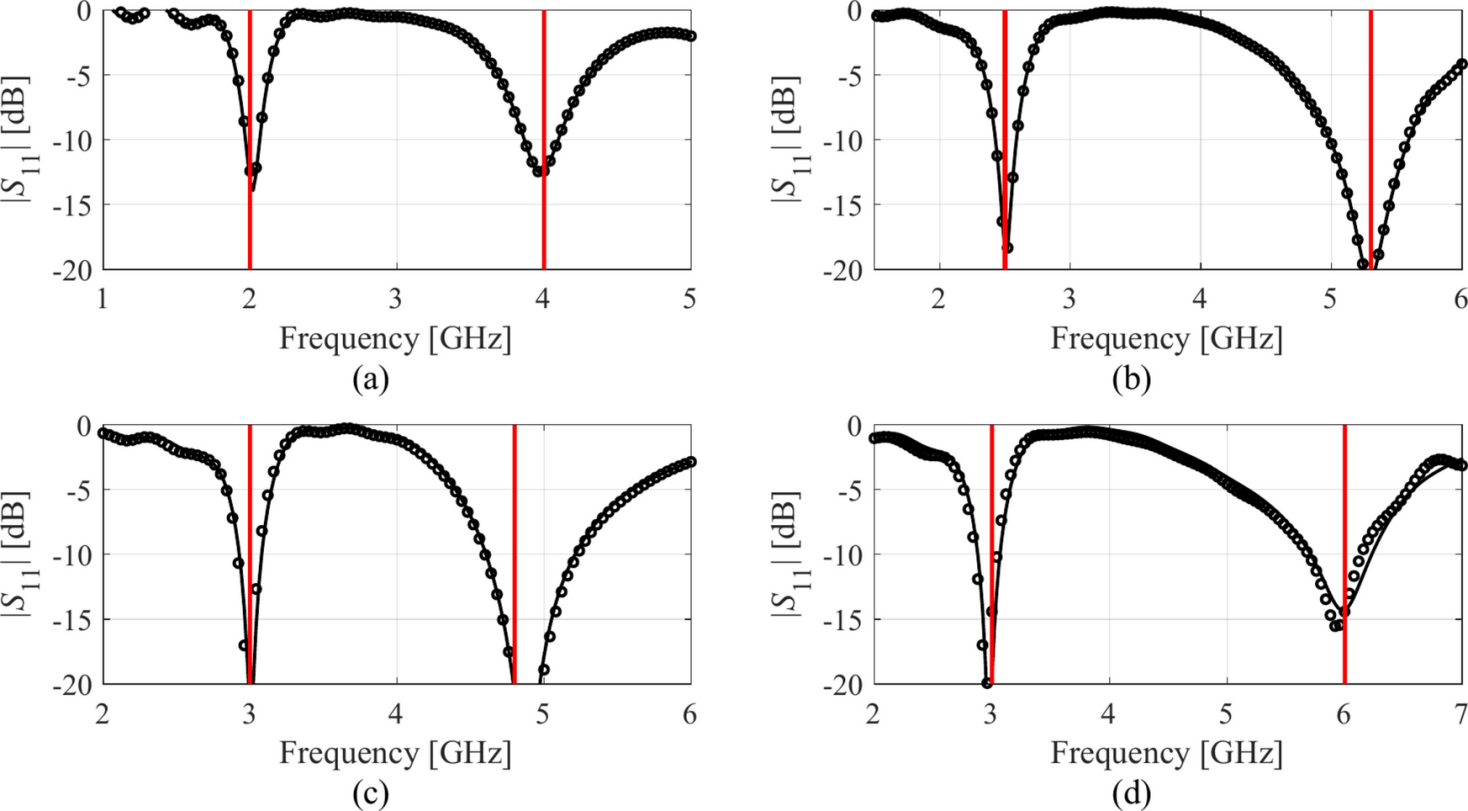
Antenna II: model-predicted |*S*_11_| at the design found by optimizing the proposed metamodel (o), and EM-evaluated characteristic (—) at the same design; model constructed for *N*_*B*_ = 800. The designs generated assuming the following specifications (lower and upper operating frequency): (**a**) *f*_1_ = 2.0 GHz, *f*_2_ = 4.0 GHz, (**b**) *f*_1_ = 2.5 GHz, *f*_2_ = 5.3 GHz, (**c**) *f*_1_ = 3.0 GHz, *f*_2_ = 4.8 GHz, (**d**) *f*_1_ = 3.0 GHz, *f*_2_ = 6.0 GHz. Vertical lines mark the target operating frequencies.

**Fig. 14:**
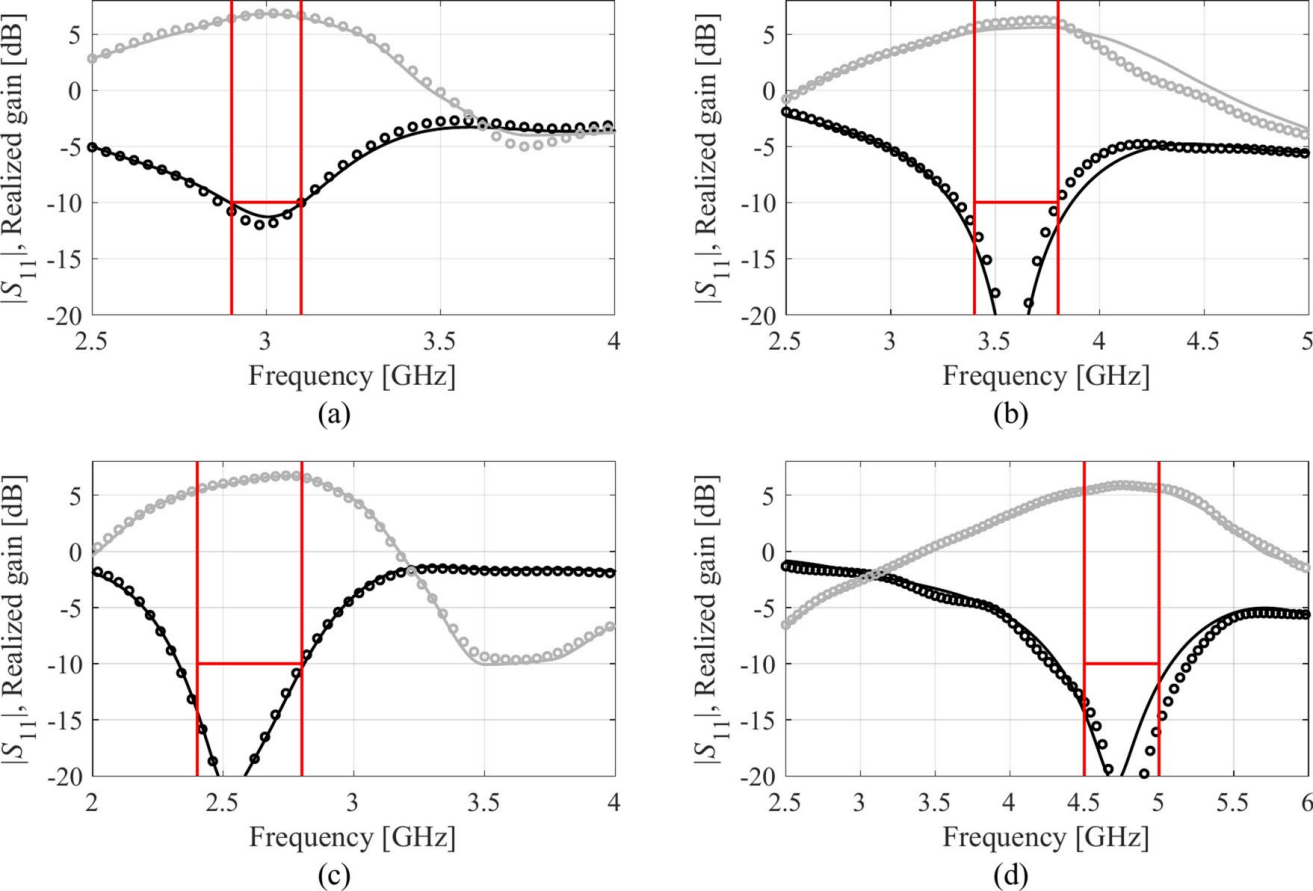
Antenna III: model-predicted |*S*_11_| (black) and realized gain (gray) at the design found by optimizing the proposed metamodel (o), and EM-evaluated characteristic (—) at the same design; model constructed for *N*_*B*_ = 800. The designs generated assuming the following specifications (lower and upper operating frequency): (**a**) *F* = [2.9 3.1] GHz, *ε*_*r*_ = 3.5, (**b**) *F* = [3.4 3.8] GHz, *ε*_*r*_ = 3.0, (**c**) *F* = [2.4 2.8] GHz, *ε*_*r*_ = 4.3, (**d**) *F* = [4.5 5.0] GHz, *ε*_*r*_ = 2.5. Vertical and horizontal lines mark the target operating band.

**Fig. 15:**
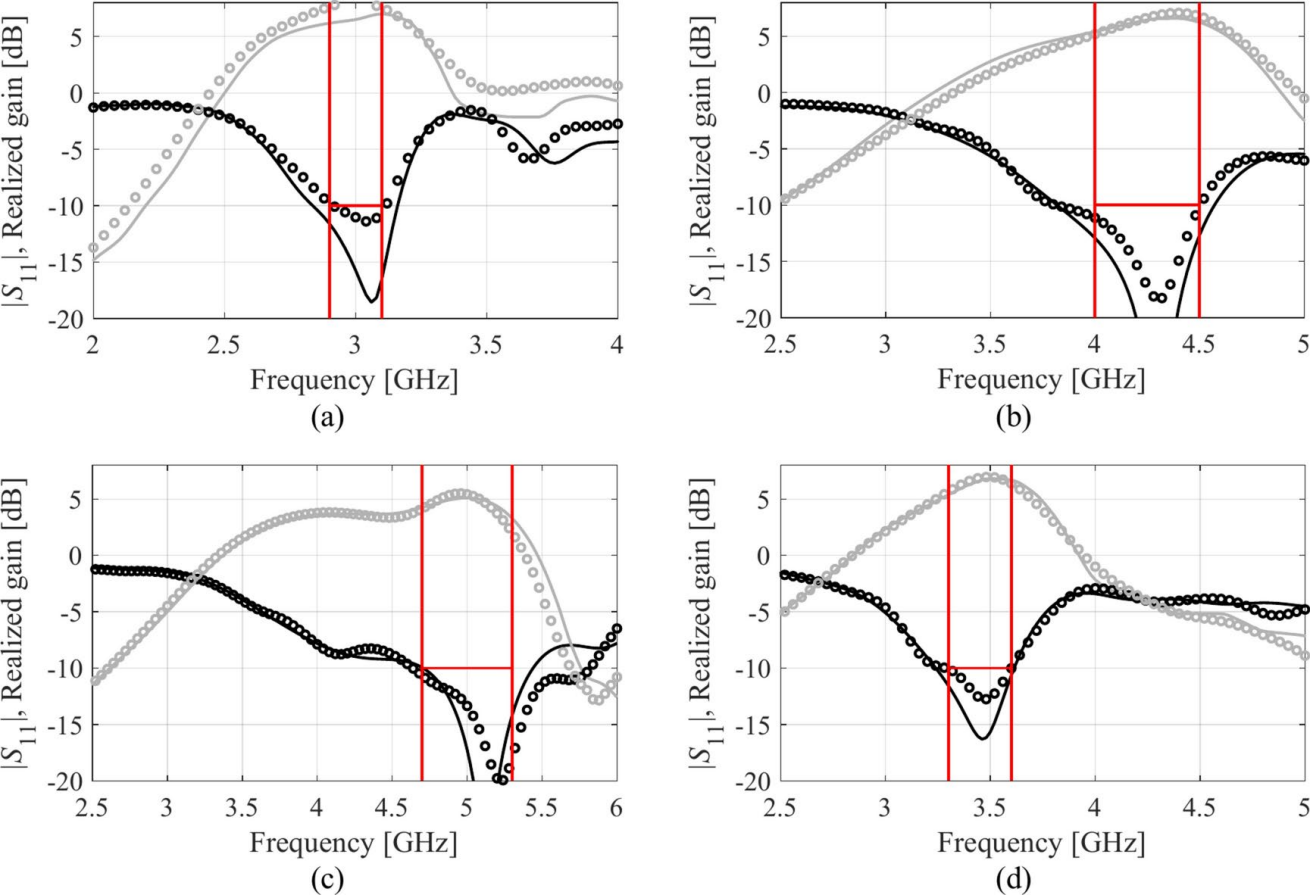
Antenna IV: model-predicted |*S*_11_| (black) and realized gain (gray) at the design found by optimizing the proposed metamodel (o), and EM-evaluated characteristic (—) at the same design; model constructed for *N*_*B*_ = 800. The designs generated assuming the following specifications (lower and upper operating frequency): (**a**) *F* = [2.9 3.1] GHz, *ε*_*r*_ = 2.5, (**b**) *F* = [4.0 4.5] GHz, *ε*_*r*_ = 2.5, (**c**) *F* = [4.7 5.3] GHz, *ε*_*r*_ = 2.0, (**d**) *F* = [3.3 3.6] GHz, *ε*_*r*_ = 3.5. Vertical and horizontal lines mark the target operating band.

Figures 12, 13, 14, and 15 show the optimized responses of Antennas I through IV, obtained through surrogate model optimization. The same images depict the EM-simulated antenna responses across the respective designs. It is evident that all specifications have been consistently fulfilled, ensuring the intended operational bandwidths are maintained. Furthermore, the correlation between the antenna characteristics generated via the surrogate model and those acquired through EM analysis is deemed satisfactory. These demonstrate the proposed model’s practical utility, particularly its ability to design antennas over wide ranges of frequencies and material parameters (here, substrate permittivity).

The discrepancy between surrogate model prediction and EM analysis is larger for Antennas III and IV than for the first two structures. This is because Antennas III and IV are extremely challenging test cases (eleven and fifteen parameters including substrate permittivity), much more complex than what is typically presented in the literature. Considering this, the accuracy of the surrogate models constructed using our approach is remarkably good (6.8% and 11.2% for Antenna III and IV, when using 800 and 1,600 training samples, respectively). The misalignment between model prediction and EM analysis for the mentioned error values is good and practically acceptable. At the same time, it should be stressed that conventional modeling methods (represented by models constructed in the original parameter space *X*, cf. Table 4) are dramatically worse (31.8% and 40.3% for Antenna III and IV, respectively, when using 800 and 1600 training samples), therefore entirely useless as design tools. Consequently, the results presented in Figs. 14 and 15 can be considered as highly successful.

### Domain dimensionality analysis

**Fig. 16:**
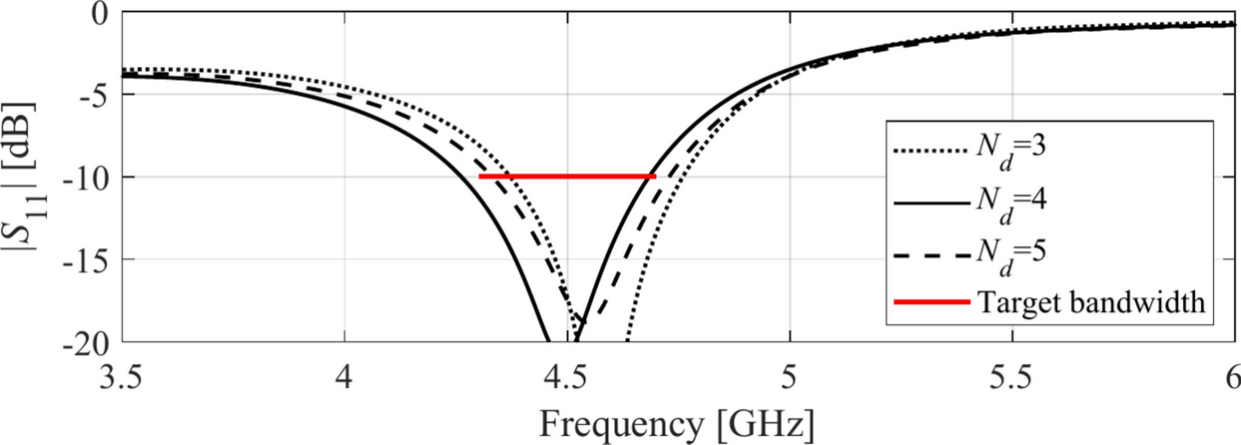
Optimization results of Antenna I for the first design scenario considered in Section "Application case studies" (*F* = [4.3 4.7], *ε*_*r*_ = 3.5). Note that the EM-simulated antenna responses obtained for *N*_*d*_ = 3 and *N*_*d*_ = 5 are noticeably worse than those obtained for *N*_*d*_ = 4.

The threshold parameter *C*_min_ has been set to the value of 0.9 in this study, meaning that the surrogate domain is spanned directions that are collectively responsible for at least 90% of antenna response changes. Clearly, increasing the value of *C*_min_ corresponds to a more strict condition, which would lead to increasing the domain dimensionality and, consequently, make the modeling process more challenging. Effectively, the model accuracy is expected to be reduced given the same number of training samples. When *C*_min_ is reduced, so is the dimensionality of the domain. The result would improve the model predictive power (again, given the same number of training samples). This has been illustrated in Table 10 for Antenna I. Note that because the eigenvalues is a discrete set, the value of ${{\sqrt {\sum\nolimits_{j = 1}^{{N_{d} }} {\lambda_{j}^{2} } } } \mathord{\left/ {\vphantom {{\sqrt {\sum\nolimits_{j = 1}^{{N_{d} }} {\lambda_{j}^{2} } } } {\sqrt {\sum\nolimits_{j = 1}^{n} {\lambda_{j}^{2} } } }}} \right. \kern-0pt} {\sqrt {\sum\nolimits_{j = 1}^{n} {\lambda_{j}^{2} } } }}$ does not change continuously. The table considers three choices of the domain dimensionality, *N*_*d*_ = 3, 4, and 5, the middle one corresponding to what was presented in Tables 3 and 4. The first and the third values are smaller and larger than the dimensionality associated with *C*_min_ = 0.9. As can be observed, reducing the dimensionality improves the surrogate’s predictive powers, whereas increasing it makes the RMS error larger. At this point, it should be noted, however, that reducing the dimensionality also reduces the domain volume, which is detrimental to the design utility of the surrogate. In particular, the optimum designs corresponding to particular specifications might not be allocated within the domain. On the other hand, increasing the dimensionality also compromises design utility, this time due to the inferior quality of the surrogate. This has been illustrated in Fig. 16 for one of the design scenarios considered in Section "Application case studies" (*F* = [4.3 4.7], *ε*_*r*_ = 3.5). Either increasing or decreasing the dimensionality leads to a slightly degraded optimization outcome. At the same time, it should be noted that the modeling process is relatively insensitive to the selection of *C*_min_. For example, for Antenna I, *N*_*d*_ = 4 is obtained for any *C*_min_ within the range 0.88 to 0.97.

**Table 10:** Antenna I: modeling results in RGSA-confined domain vs domain dimensionality.

Number of training points	Modeling error (reduced domain *X*_*d*_)		
	*N*_*d*_ = 3 [*C*_min_ = 0.88]	*N*_*d*_ = 4 [*C*_min_ = 0.94]	*N*_*d*_ = 5 [*C*_min_ = 0.0.97]
50	19.7%	49.1%	53.1%
100	10.2%	18.3%	27.3%
200	5.3%	6.4%	9.1%
400	3.5%	3.9%	6.8%
800	3.2%	3.5%	5.5%

An additional analysis is carried out for Antenna I concerning the number *N*_*s*_ of random observables utilized to carry out the sensitivity analysis (cf. Section "Rapid global sensitivity analysis"). The verification part of the paper was executed using *N*_*s*_ = 50. Table 11 shows the spectral analysis results of the relocation matrix ***S*** obtained using *N*_*s*_ = 100 and *N*_*s*_ = 200. As can be observed, the progression of the normalized eigenvalues is quite similar in all cases. This suggests that RGSA is relatively insensitive to the choice of *N*_*s*_. Nonetheless, as mentioned in Section "Modelling procedure", it is generally recommended to increase *N*_*s*_ with the number of design variables, which is also suggested in the literature concerning GSA (e.g.^98–103^).

**Table 11:** Normalized eigenvalues of the relocation matrix ***S*** for different values of *N*_*r*_*.*

*N*_*s*_	Normalized eigenvalues								
	*λ*_1_	*λ*_2_	*λ*_3_	*λ*_4_	*λ*_5_	*λ*_6_	*λ*_7_	*λ*_8_	*λ*_9_
50	1.00	0.74	0.63	0.48	0.35	0.29	0.23	0.17	0.06
100	1.00	0.60	0.52	0.42	0.30	0.27	0.19	0.16	0.11
200	1.00	0.58	0.46	0.34	0.28	0.25	0.19	0.16	0.10

## Conclusion

This article introduces an innovative approach to computationally efficient behavioral modeling of antennas. Our methodology leverages a rapid global sensitivity analysis (RGSA) procedure to identify the essential directions within the design variable space, significantly impacting antenna response variability. The surrogate model’s region of validity is then determined using a limited subset of these critical directions, determined through appropriate response variability indicators. This dimensionality reduction substantially enhances predictive power and enables achieving acceptable error levels even when using a restricted number of training points. Importantly, the surrogate maintains sufficient flexibility in representing antenna characteristics variability, ensuring that the model’s design utility remains intact. The efficiency of the introduced method has been comprehensively validated across four antenna structures, considering modeled responses such as reflection coefficient and realized gain over wide frequency ranges (typically between 1 and 7 GHz) and relative permittivity of the substrate (typically between 2.0 and 5.0). The RGSA-based domain definition results in remarkably accurate predictive power (relative RMS error within a few percent) despite challenging parameter space configurations where conventional modeling methods fail. Moreover, the proposed models are successfully employed for antenna optimization across various scenarios, including matching improvement over target frequency bands, gain maximization, and different combinations of target bands and substrate permittivity values. These findings suggest that the presented approach provides an attractive alternative to available modeling methods, especially for building low-cost, design-ready replacement models. The procedure is generic compared to some recently proposed methods, such as performance-driven modeling approaches, and is relatively straightforward to implement.

The proposed technique can be used to model input characteristics of array antennas such as microstrip patch arrays, MIMO antennas, and so on. From the perspective of the modeling process, the only difference is that the number of responses to be modeled would be larger (i.e., equal to the number of array excitation points), similarly as modeling of Antennas III and IV involved representing of the reflection and realized gain responses. This does not bring any fundamental limitations to the proposed methodology. At the same time, modeling of other types of responses is also possible (e.g., directivity as a function of array geometry parameters), although direct modeling of radiation patterns is a considerably more complex matter, which will be addressed in future work. It should be mentioned that although the proposed methodology has been applied to microstrip antennas, it is more generic. The reason is that none of the modeling steps (including sensitivity analysis, dimensionality reduction, surrogate model definition, or model identification) are related to the specific properties of antenna responses. Thus, the method can be applied to other types of high-frequency systems or even components/devices within other engineering disciplines. One of the topics of future work will be to demonstrate our approach’s applicability to other microwave and antenna structures classes.

## Data Availability

The datasets used and/or analyzed during the current study available from the corresponding author on reasonable request.
